# Closely related viruses of the marine picoeukaryotic alga *Ostreococcus lucimarinus* exhibit different ecological strategies

**DOI:** 10.1111/1462-2920.14608

**Published:** 2019-05-13

**Authors:** Amy E. Zimmerman, Charles Bachy, Xiufeng Ma, Simon Roux, Ho Bin Jang, Matthew B. Sullivan, Jacob R. Waldbauer, Alexandra Z. Worden

**Affiliations:** ^1^ Monterey Bay Aquarium Research Institute Moss Landing CA USA; ^2^ Department of the Geophysical Sciences University of Chicago Chicago IL USA; ^3^ Department of Microbiology Environmental and Geodetic Engineering, The Ohio State University Columbus OH USA; ^4^ Department of Civil Environmental and Geodetic Engineering, The Ohio State University Columbus OH USA; ^5^ Ocean EcoSystems Biology Unit, Marine Ecology Division GEOMAR Helmholtz Centre for Ocean Research Kiel Kiel DE

## Abstract

In marine ecosystems, viruses are major disrupters of the direct flow of carbon and nutrients to higher trophic levels. Although the genetic diversity of several eukaryotic phytoplankton virus groups has been characterized, their infection dynamics are less understood, such that the physiological and ecological implications of their diversity remain unclear. We compared genomes and infection phenotypes of the two most closely related cultured phycodnaviruses infecting the widespread picoprasinophyte *Ostreococcus lucimarinus* under standard‐ (1.3 divisions per day) and limited‐light (0.41 divisions per day) nutrient replete conditions. OlV7 infection caused early arrest of the host cell cycle, coinciding with a significantly higher proportion of infected cells than OlV1‐amended treatments, regardless of host growth rate. OlV7 treatments showed a near‐50‐fold increase of progeny virions at the higher host growth rate, contrasting with OlV1's 16‐fold increase. However, production of OlV7 virions was more sensitive than OlV1 production to reduced host growth rate, suggesting fitness trade‐offs between infection efficiency and resilience to host physiology. Moreover, although organic matter released from OlV1‐ and OlV7‐infected hosts had broadly similar chemical composition, some distinct molecular signatures were observed. Collectively, these results suggest that current views on viral relatedness through marker and core gene analyses underplay operational divergence and consequences for host ecology.

## Introduction

The structure and function of marine ecosystems are profoundly influenced by the activity of viruses. Upon infection, viruses can alter the physiology of individual cells by manipulating host metabolism to support requirements for viral replication (Puxty *et al.,* 2016; Rosenwasser *et al.,* 2016; and references therein). Viral infection and lysis of specific bacterial host genotypes leads to changes in the size and genetic makeup of host populations and thus appear to represent a significant selective pressure driving host evolution as well, although difficult to confirm in the marine environment (Martiny *et al.,* 2014; Thingstad *et al.,* 2014). Furthermore, the consequences of viral infection at the population level are generally considered to scale up to influence community composition and nutrient availability to the broader community via lysis products and/or changes in host metabolism (Fuhrman, 1999; Suttle, 2007; Weitz and Wilhelm, 2012; Hamblin *et al.,* 2014; Ma *et al.,* 2018). Much of the above and other aspects of our mechanistic understanding of virus–host interactions in the marine realm comes from bacteriophages and cyanophages (e.g., Breitbart *et al.,* 2018), despite the important roles of eukaryotic phytoplankton and their viruses in marine ecosystems. The dynamics of viruses infecting unicellular eukaryotic phytoplankton are perhaps best known for the coccolithoviruses isolated against *Emiliania huxleyi* (Wilson *et al.,* 2009; Short, 2012; Nissimov *et al.,* 2017). Thus, although the general ecological importance of marine phytoplankton (both eukaryotic and cyanobacterial) and their viruses is widely recognized (e.g., Middelboe and Brussaard, 2017), many fundamental first‐order questions persist about how viruses interact with phytoplankton hosts, how host physiology impacts viral production and how these factors impact broader biogeochemical cycles (Brum and Sullivan, 2015; Breitbart *et al.,* 2018; Horas *et al.,* 2018).

The first reported dsDNA virus infecting a marine eukaryotic alga was MpV, isolated from the picoeukaryotic alga *Micromonas pusilla* (Mayer and Taylor, 1979), a member of the diverse prasinophyte algae. Although picoeukaryotes (< 2 or 3 μm cell diameter) are less abundant than cyanobacteria in many marine settings, they contain more biomass per cell (6.5–14 times more carbon) and can grow at higher rates in the wild (Worden *et al.,* 2004; Cuvelier *et al.,* 2010). Thus, even in systems where their abundance is lower than cyanobacteria, they can dominate the total local primary production in the picoplanktonic size fraction and contribute as much as 79% of the carbon consumed by higher trophic levels in some systems (Li, 1994; Worden *et al.,* 2004). Viruses have also been estimated to lyse between 9% and 25% of the standing stock of picoeukaryotic phytoplankton population biomass daily in the surface waters of the North Sea during summer (Evans *et al.,* 2003; Baudoux *et al.,* 2008). Most marine eukaryotic phytoplankton viruses known to date, including MpV, belong to the *Phycodnaviridae*, a family of large double‐stranded DNA viruses that infect eukaryotic algae (Chen and Suttle, 1996), within the Nucleocytoplasmic Large DNA Viruses (NCLDV) (Yutin *et al.,* 2009). Other phycodnaviruses include *Paramecium bursaria Chlorella* Virus 1 (PBCV‐1, genus *Chlorovirus*), *Emiliania huxleyi* virus 86 (EhV‐86, a *Coccolithovirus*) and *Chrysochromulina brevifilum* virus PW1 (CbV‐PW1, a *Prymnesiovirus*) (Van Etten *et al.,* 2002; Wilson *et al.,* 2009) and thus infect a broad phylogenetic range of host organisms.

Dynamics of the phycodnavirus MpV‐SP1, which also infects *Micromonas pusilla* (Cottrell and Suttle, 1991), and MpV‐08T, a prasinovirus that infects *M. commoda*‐like isolate LAC38 (Maat *et al.,* 2014), have been studied under various conditions. It has been shown that their latent period and viral production levels are impacted by phosphate availability (Maat *et al.,* 2014, 2016; Bachy *et al.,* 2018) as well as nitrate availability (Maat and Brussaard, 2016). However, these studies were unable to dissect whether the results were due to changes in host growth rate (i.e., indirect effect) or the actual growth limiting factor (i.e., direct effect). This is important because prior studies have inferred that phosphate availability in particular plays a direct role in viral dynamics and production, yet experimental evidence is sparse. One advantage of using photosynthetic microbes as an experimental system is that energy production can be manipulated by light availability, facilitating control of host growth rate. Previous studies that investigated the influence of light on virus–host interactions in *Micromonas* species (Baudoux and Brussaard, 2008; Maat *et al.,* 2016; Piedade *et al.,* 2018) found support for an impact of irradiance level on latent period and viral production only with a concurrent shift in host growth rate. It remains unclear whether the magnitude of change in host growth rate needed to elicit an impact on the viral life cycle is unique to each host and/or virus, or whether there is a common threshold across host species. Regardless, manipulation of light availability is an effective tool for controlling host growth physiology in phytoplankton.

Several other phycodnaviruses have been identified that infect picoprasinophyte relatives of *Micromonas*, especially *Ostreococcus* (Courties *et al.,* 1994; Worden *et al.,* 2004). *Micromonas* and *Ostreococcus* differ in the latter being smaller, non‐flagellated and restricted to tropical through temperate environments. *Ostreococcus tauri* (Clade C, Guillou *et al.,* 2004), isolated from an oyster lagoon and having a very limited marine distribution (Courties *et al.,* 1994; Demir‐Hilton *et al.,* 2011), is now often used as a model system for interrogating virus–host interactions (Thomas *et al.,* 2011; Heath and Collins, 2016; Heath *et al.,* 2017). In marine ecosystems, quantitative enumeration of Clade OI (*sensu* Demir‐Hilton *et al.,* 2011; akin to Clade A, *sensu* Guillou *et al.,* 2004) shows it can be highly abundant and is widespread in coastal and mesotrophic environments (Demir‐Hilton *et al.,* 2011; Simmons *et al.,* 2016; Clayton *et al.,* 2017; Limardo *et al.,* 2017). This clade is represented by the genome‐sequenced species *O. lucimarinus* (Worden *et al.,* 2004; Palenik *et al.,* 2007), is more broadly distributed than *O. tauri* (Simmons *et al.,* 2016), and viruses infecting it have been isolated from geographically distant coastal sites (Bellec *et al.,* 2010; Derelle *et al.,* 2015). The *O. lucimarinus* viruses and their cultured host provide the opportunity to develop an ecologically relevant eukaryote–virus model system, that will extend understanding of naturally occurring virus–host interactions from the well‐studied coccolithoviruses and *Micromonas* viruses and their respective hosts to another key eukaryotic marine alga.

To better understand the significance of viral diversity, comparative studies are needed that scrutinize the dynamics of different viruses that infect a common host (Zingone *et al.,* 2006; Nissimov *et al.,* 2013, 2016). Unfortunately, comparisons between studies are often faulty due to differences in experimental parameters that directly impact infection dynamics. Such parameters include encounter rates, proportion of infectious virions, host physiological state or temporal resolution (Murray and Jackson, 1992; Brown and Bidle, 2014; Mojica and Brussaard, 2014). Moreover, viral infectivity levels are often assumed to be the same as in prior experiments or publications, and many studies do not directly test the infectivity of virions by plaque or end‐point dilution assay (Taylor, 1962; Van Etten *et al.,* 1983). Thus, parallel investigations that use the same experimental approaches, and accurately parameterize host cells and viral infectivity, are still needed and should enhance our ability to identify general principles governing virus–host interactions.

Here, we compared the infection dynamics of two phycodnaviruses of the picoprasinophyte alga *Ostreococcus lucimarinus* CCMP2972 (CCE9901) (Worden *et al.,* 2004; Palenik *et al.,* 2007; Derelle *et al.,* 2015). Specifically, we evaluated host physiology, host population dynamics, virus life cycle and the chemistry of dissolved organic matter (DOM) generated from lysis. Several viruses infecting *O. lucimarinus* (referred to as ‘OlVs’ for *Ostreococcus lucimarinus* Viruses) have been genome sequenced and described (Derelle *et al.,* 2015). Our experiments focused on comparing the infection phenotypes of OlV1 and OlV7, both representatives of the OlV type 1 genomic subgroup, but isolated from geographically distant coastal locations (Mediterranean Sea and eastern North Pacific Ocean respectively; Derelle *et al.,* 2015). Here, parallel characterization of infection dynamics showed OlV7 is more virulent than OlV1, leading to quicker demise of the host population, whereas the replicative cycle of OlV1 is more resilient to depressed host growth rate. These findings provide evidence for the ecological/functional relevance of what might be considered fine‐scale genetic diversity in marine studies, as well as new insights into the impact of viral infection on the ecology of this prominent picophytoplankton species.

## Results

### *Viral genome comparison*

**Figure 1:**
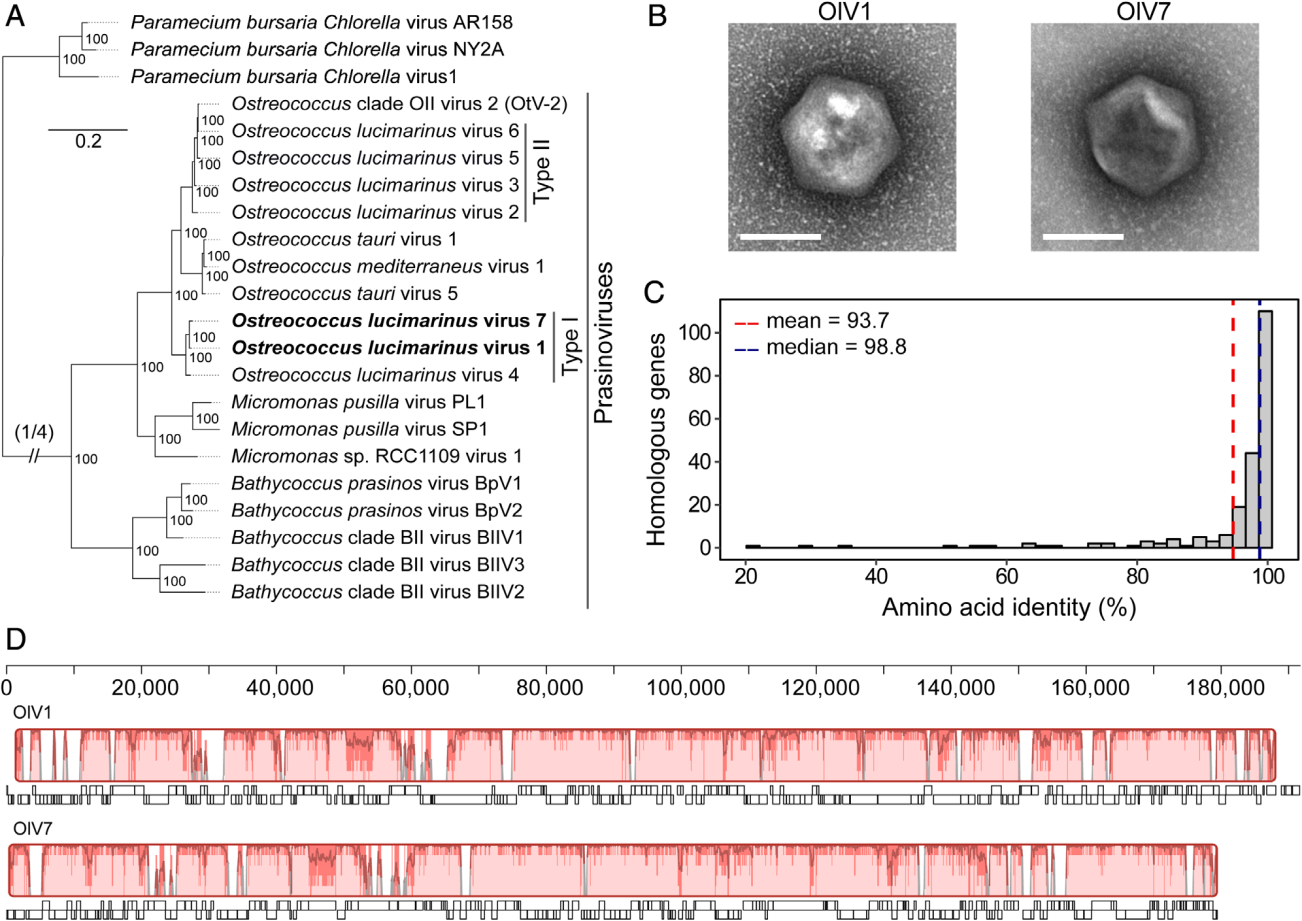
Comparisons of *Ostreococcus lucimarinus* viruses OlV1 and OlV7. A. Maximum‐likelihood phylogenetic tree of green algal viruses inferred from a concatenated amino acid alignment of 22 shared ‘core algal virus proteins’ (7001 positions). Viruses studied herein are highlighted in bold, and *Chlorovirus* were used as an outgroup (grey). Bootstrap support reflects the percent of 100 replicates. Note that OtV‐2 was originally misnamed as being an *O. tauri* virus, but was isolated against RCC393, an *Ostreococcus* Clade OII species (*sensu* Simmons *et al.,* 2015; Clade B *sensu* Guillou *et al.,* 2004). B. Transmission electron micrographs of OlV1 and OlV7 (scale bar, 100 nm). OlV1 capsids measured 146 ± 1 nm and OlV7 measured 140 ± 4 nm in diameter (*n* = 5 virions). C. A histogram of percent amino acid identity for 212 orthologous genes identified in OlV1 and OlV7 genomes by reciprocal BLAST (minimum identity of 20%, minimum coverage of 50% of the shorter sequence). The mean (93.7%, red dashed line) and median (98.8%, blue dashed line) identities are indicated. D. Synteny analysis based on the alignment of OlV1 and OlV7 annotated genome sequences. Alignment is shown relative to nucleotide position in OlV1 genome. Boxes with identical colours represent Local Colinear Blocks, indicating homologous DNA regions without sequence rearrangements. Similarity profiles (trace) show average level of conservation at each position. Annotated genes (white boxes below each genome) are shown with genes in the forward (boxes above black line) and reverse directions (boxes below black line) indicated.

Lytic phycodnaviruses OlV1 and OlV7 were initially isolated from a eutrophic northwestern Mediterranean coastal lagoon and northeastern Pacific Ocean coastal habitat respectively (Derelle *et al.,* 2015). Here, we re‐sequenced and re‐annotated the viral genomes from the newly purified OlV1 and OlV7 used in this study, to confirm their identity and explicitly compare genetic similarities between these two viruses, in addition to imaging them. They are icosahedral with similar capsid diameters of approximately 140 nm (Fig. 1B). Their genomes are similar in size to previous estimates (around 200 kb), and the genome sequences were nearly identical to the previous genome assemblies (from Moreau *et al.,* 2010; Derelle *et al.,* 2015; 97.5% genome‐wide nucleotide identity for OlV1 and 98.6% for OlV7). When applying the same annotation procedure, the number of predicted genes varied slightly between the previous genome assemblies and these re‐sequenced genomes (± 5 and 1 genes for OlV1 and OlV7 respectively, Supporting Information Table S1). Genes that were recovered from only one of the genome assemblies (i.e., missing from the other) were investigated by tblastn analyses which showed that the differences in the number of predicted protein‐encoding genes from the previous assembly/annotation methods were accounted for by (a) genes that were originally identified in terminal inverted repeat regions, which were not found in repeat after resequencing (i.e., complete or truncated sequences from terminal inverted repeat regions were found only at one terminus for resequenced OlV1 and OlV7 genomes respectively; affected three genes each for OlV1 and OlV7), (b) genes from the original annotations that were predicted to encode short proteins [<82 amino acids (aas), with start and stop codons] and were subsequently not annotated as protein‐encoding genes (one case each for OlV1 and OlV7), and (c) genes from the original annotations that were combined into one longer, continuous gene (two cases for OlV1). In the absence of further experimental evidence, it is not possible to confirm or refute these predictions. Both OlV1 and OlV7 exhibited the characteristic OlV type I subgroup 32‐kb DNA fragment that is inverted relative to its positioning in the OlV type II subgroup.

Gene synteny between the re‐sequenced OlV1 and OlV7 is preserved across much of the length of the genomes (Fig. 1D). This is supported by high sequence identity throughout much of the genomes (shown as the trace in Fig. 1D) and arrangement of the genomes into one local colinear block (i.e., homologous DNA region without sequence rearrangements). Interspersed regions contained lineage‐specific segments that were not aligned, housing 29 and 21 genes specific to OlV1 and OlV7 respectively, although orthologs of these genes are found in some other sequenced prasinovirus genomes (as below).

Whole‐genome reciprocal protein BLAST and orthology analyses show that OlV1 and OlV7 share 215 and 218 protein‐encoding genes, respectively, comprising 212 ortho‐groups and representing 88%–91% of each virus' predicted gene set (Supporting Information Table S2). Proteins within most orthogroups have high average aa identity (93.7% ± 14.7%, Fig. 1C), whereas members of 12 had aa identities below 70% (40.2% ± 19.6% average aa identity for this subset). When available, domain analyses indicated that these low‐identity orthologues have similar putative functions. Most of the predicted viral genes encode proteins of unknown function (i.e., 148 of 212 orthologous sequences lack discernable Pfam domains, *E*‐value <10^−5^). Twenty‐nine protein‐encoding genes in OlV1 and 21 in OlV7 were not shared with the other, and for two and four of these respectively, no significant blastp hits were returned. Orthologues were identified in other prasinovirus genomes for all but three of the OlV1‐ and two of the OlV7‐specific genes (Supporting Information Table S3). Most of OlV1‐ and OlV7‐specific genes lack recognizable functional domains (Supporting Information Table S3). Putative functions could be assigned to 11 of the 29 OlV1‐specific predicted proteins, whereas only four of the 21 OlV7‐specific proteins matched domains with putative functions (Supporting Information Table S3). Among those with putative functions, OlV7 encodes six possible Fe(II)/2‐oxoglutarate‐dependent oxygenase proteins which belong to three different families (Supporting Information Tables S2 and S3). One protein/family is absent from OlV1 but found in OlV7 and five other viruses that infect *Ostreococcus*, whereas the other two families, comprised of five of the six putative OlV7 oxygenase proteins, have orthologs in OlV1 (Supporting Information Table S2). The OlV1 unique genes included a triacylglycerol lipase and a putative rhamnose synthetase, with rhamnose being found in the outward‐facing glycan portions of PBCV‐1 virion capsids (Wang *et al.,* 1993; Wilson *et al.,* 2009). OlV1 also encodes a predicted 3‐dehydroquinate synthase protein likely involved in biosynthesis of aromatic aas, as well as a putative NADH‐enoyl acyl carrier protein reductase used in fatty acid biosynthesis in *Escherichia coli* (Magnuson *et al.,* 1993). Thus, the two viruses share many genes but also have sets of non‐homologous functions.

### *Optimizing reproducibility of experimental conditions*

Many one‐step growth experiments synchronize viral infection by post‐adsorption dilution of unadsorbed virions to characterize latent period and burst size (Hyman and Abedon, 2009). Here, preliminary experiments showed that large‐scale dilution (1:100) resulted in a depressed growth rate of *O. lucimarinus* compared to undiluted cultures. Therefore, we adopted a setup that differed from typical one‐step viral infection experiments in that no dilution was performed following the initial adsorption period. Thus, in our study, the infection dynamics observed may not be the result of one synchronized infection event because some degree of reinfection may have occurred over the experimental time course. However, each experiment was initiated for OlV1 and OlV7 in parallel with equivalent starting conditions to evaluate their infection phenotypes and consequences for host physiology and population dynamics. Controlled factors included host density, host growth phase, culture volume and target ratio of infectious virions to host cells.

Preliminary experiments also indicated that OlV7 produced a greater proportion of infectious progeny than OlV1. For each experiment, we used Most Probable Number (MPN) assays to quantify the number of virions capable of entering host cells and completing an infection cycle. An endpoint dilution method was used because this planktonic host grew poorly on solid and soft agar media, as also observed for its relative *M. pusilla* (Waters and Chan, 1982). Therefore, a plaque assay akin to that used for *O. tauri* (Derelle *et al.,* 2008) could not be established. The MPN assays were performed before the experiment, and again during the experiment, to verify infectivity of the inocula used. The latter showed that infectivity of the OlV1 stock was 14% (95% confidence interval, CI95 = 8%–23% over 24 replicate wells for each dilution level), whereas infectivity of the OlV7 stock was 32% (CI95 = 20%–52%). In our final experiment, the measured infectivity of the OlV7 inoculum was lower than expected from preliminary experiments, thus the OlV1 experimental treatments had a slightly higher ratio of infectious virions to host cells (multiplicity of infection, MOI 3.4, Table 1) than the OlV7 treatments (1.5–1.7).

**Table 1:** Measured infection parameters for *Ostreococcus lucimarinus* viruses 1 and 7.

	OlV1		OlV7	
Parameter	SL^a^	LL^b^	SL	LL
Infectivity^c^	14% (7.9%–23%)	14% (7.9%–23%)	32% (20%–52%)	32% (20%–52%)
Virus:host	25 ± 1	25 ± 2	4.8 ± 0.3	5.5 ± 0.1
MOI (95% CI)	3.4 (2.0–5.6)	3.4 (1.9–5.5)	1.5 (1.0–2.5)	1.7 (1.1–2.8)
Latent period (h)	4.5*–8.5	6.5–8.5	6.5–8.5	8.5–10.5
Burst size^d^	435 ± 251	237 ± 47	682 ± 408	51 ± 20

a SL = 105–115 μmol photons m^−2^ s^−2^ irradiance.

b LL = 15 μmol photons m^−2^ s^−2^ irradiance.

c Proportion of virus population capable of entering host cells and producing progeny.

d Estimated from the increase in free viruses and the loss of host cells between 18.5 and 24.5 h after addition of virus (*n* = 3). Units are progeny virions per cell.

* Not significantly different from zero (Welch's one‐sample *t*‐test for mu = 0, *P* = 0.25), but likely due to variation among replicates, so plausible that burst started to occur before this time point.

### *Host growth and cell cycle dynamics*

**Figure 2:**
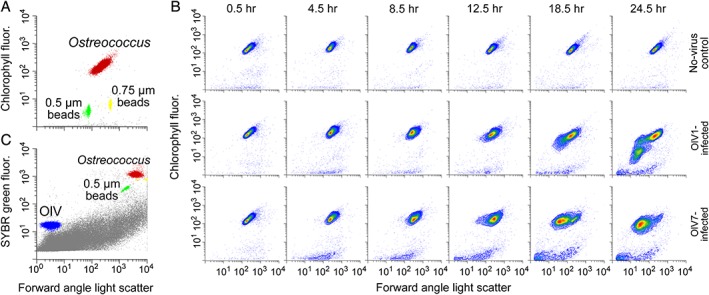
Representative flow cytometry plots. A. Discrimination of *O. lucimarinus* host populations based on chlorophyll autofluorescence (692 nm) versus forward angle light scatter (FALS). B. Discrimination of OlV virus populations based of green (SYBR) fluorescence (520 nm) versus FALS. The host population (red) and virus population (blue) are shown along with beads used for normalization (yellow and green) and background events (grey, possibly cell debris or media components). C. Chlorophyll fluorescence versus FALS over the infection cycle for non‐infected control, OlV1‐infected, and OlV7‐infected cultures acclimated to 105–115 μmol photons m^−2^ s^−2^ irradiance (SL). Note that both *x*‐ and *y*‐axes are plotted on a log scale.

**Figure 3:**
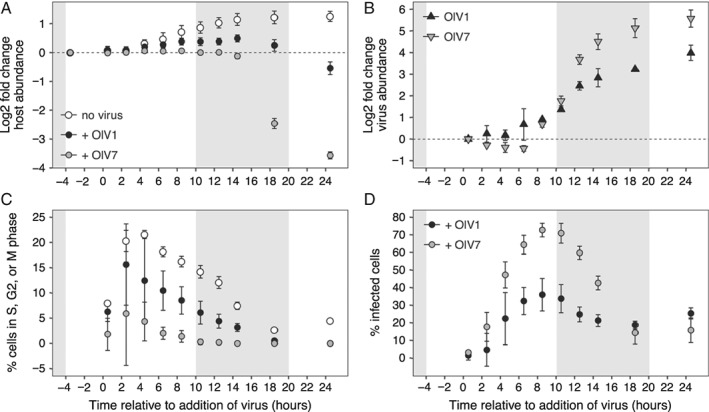
Growth of host cultures acclimated to 105–115 μmol photons m^−2^ s^−2^ irradiance and viral life cycle of OlV1 and OlV7 resolved by analytical flow cytometry. A. Growth curves of algal hosts without viruses (open circles) and with addition of OlV1 (black circles) or OlV7 (grey circles), shown as the log2 fold change in abundance (equivalent to number of generations during exponential growth) since dawn (T‐4 h). B. OlV1 (black triangles) and OlV7 (grey triangles) abundance over the infection cycle shown as the log2 fold change relative to the time viruses were added to cultures (*T* = 0 h). C. Percentages of algal cells that were actively dividing (sum of cells in S, G2 or M phases) as inferred from cell cycle analysis of SYBR‐stained samples. The growth of OlV1‐ and OlV7‐infected cultures relative to non‐infected cultures in panel A was used to calculate the percentages of dividing cells in infected cultures at each time point from non‐infected culture values (see methods for more details). D. The percentages of infected host cells were inferred from SYBR‐stained samples, after accounting for cells in S, G2 and M phases of the cell cycle. Points show mean ± standard deviation of biological replicates (*n* = 3). Shaded areas indicate dark period in 14:10 h diel cycle.

The impact of infection by OlV1 or OlV7 on host population dynamics (i.e., growth and mortality) was assessed by comparing temporal changes in host abundance and cell cycle phase using flow cytometry (Fig. 2). Non‐infected host cultures doubled more than once over the course of the experiment, having a growth rate of 0.76 ± 0.11 day^−1^ under Standard Light (‘SL’) conditions (Table 2), consistent with measured growth rates of *O. lucimarinus*‐containing picoeukaryote populations in the eastern North Pacific Ocean near San Diego, CA, USA (Worden *et al.,* 2004). A significant effect of virus treatment on host abundance was detected by 6.5 h after addition of OlV7 (*P* < 0.05), which was earlier than for OlV1‐infected treatments (10.5 h, *P* < 0.02), and the differences remained significant to the end of the experiment for both viruses. Furthermore, unlike the controls and OlV1 treatments, the abundance of hosts in OlV7‐infected treatments did not increase significantly over the first 14.5 h of the experiment, and only changed significantly at the point where considerable cell lysis was apparent (Fig. 3A; 18.5 h, *P* < 0.001). Thus, OlV1 and OlV7 differentially affect growth of their host such that cultures infected by OlV1 continue dividing for several hours after addition of virus, whereas OlV7 arrests host growth and results in a much larger fraction of host mortality within 24 h.

**Table 2:** Host mean specific population growth rate (d^−1^) over the experiment^a^. Note that negative values indicate the rate of mortality (resulting in cell loss) was higher than growth.

	SL^b^	LL^c^	Welch's *t*‐test
No virus	0.76 ± 0.11	0.25 ± 0.05	*P* < 0.01
+OlV1	−0.33 ± 0.13	−0.57 ± 0.06	*P* = 0.07 (NS)
+OlV7	−2.2 ± 0.1	−1.4 ± 0.1	*P* < 0.005

a Calculated for interval from the beginning (−3.5 h) to the end (24.5 h) of the experiment (*n* = 3).

b SL = 105‐115 μmol photons m^−2^ s^−2^ irradiance.

c LL = 15 μmol photons m^−2^ s^−2^ irradiance.

*Ostreococcus lucimarinus* exhibited clear cell‐cycle synchronization in non‐infected controls, with division beginning 6–8 h into the light phase and continuing until near dawn under SL conditions (Supporting Information Fig. S1A). However, amendment with viruses significantly reduced (*P* < 0.05) the proportion of host cells in S, G2 or M cell cycle phases (i.e., non‐G1 phase) at all but the 2.5 h time point (Fig. 3C). Specifically, OlV7‐infected treatments had a significantly lower percentage of host cells in S, G2 or M phases than non‐infected cultures at all time points (*P* < 0.05), with the exception of the samples taken at 2.5 h. Significance in the 2.5 h time point was not observed due to variation among biological replicates (*P* > 0.1). OlV1‐infected treatments followed the cell cycle dynamics of non‐infected cultures relatively closely early in the experiment, diverging only after several hours (6.5 h, *P* < 0.02). In addition, the percentage of cells in S, G2 or M phases differed significantly between OlV1‐ and OlV7‐infected treatments during the middle of infection (from 6.5 to 14.5 h after addition of virus, *P* < 0.02). These results demonstrate that while both viruses disrupted the host cell cycle, infection by OlV7 stops progression of the cell cycle beyond G1 phase and causes more rapid and complete demise of the host population than OlV1 infection under the conditions tested (Fig. 3A, C).

### *Influence of host growth on infection dynamics*

We next asked how growth rate of the host might influence the observed differences between OlV1 and OlV7 host dynamics and kinetics during infection. To this end, we compared the SL infection results to those in Limited Light (‘LL’) host cultures. The host cultures grew at 0.76 ± 0.06 day^−1^ and 0.091 ± 0.082 day^−1^ before infection respectively (all treatments included, *n* = 9). The magnitude of differences between OlV1 and OlV7 treatments were diminished in LL as compared to SL conditions for several metrics including log2 fold change host abundance (Fig. 3A, Supporting Information Fig. S2A), proportion of host cells in S, G2 or M cell cycle phases (Fig. 3C, Supporting Information Fig. S2C) and log2 fold change free virus abundance (Fig. 3B, Supporting Information Fig. S2B). However, OlV7 again inhibited cell division earlier (Supporting Information Fig. S2C) and caused more rapid cell lysis than OlV1 (Supporting Information Fig. S2A), demonstrating its greater virulence.

**Figure 4:**
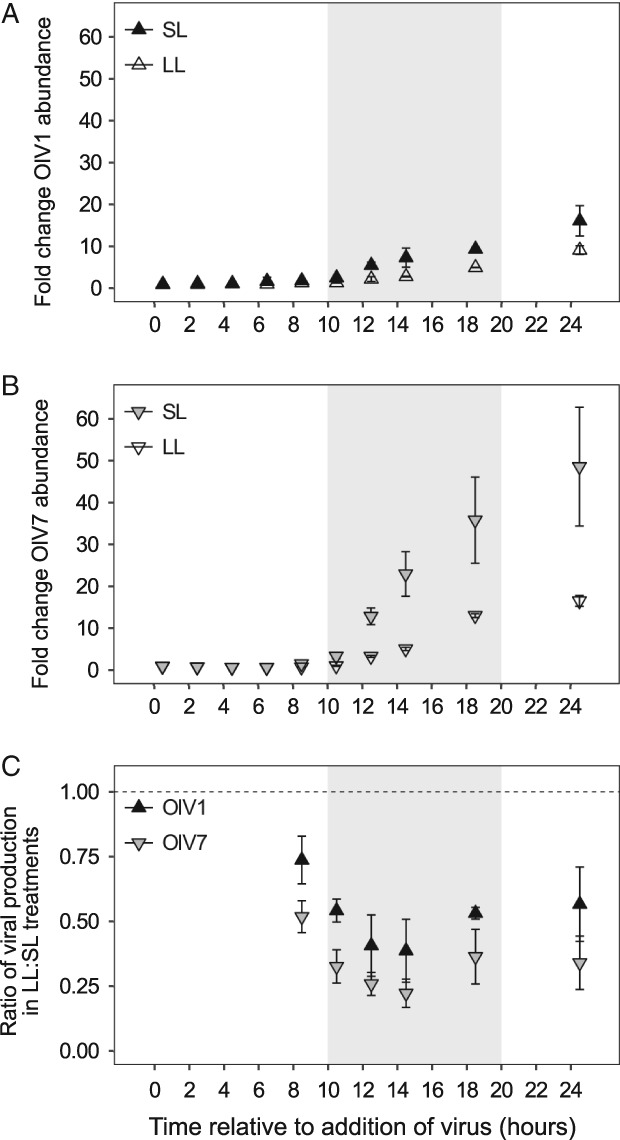
Response of viral production to host cultures acclimated to 105–115 μmol photons m^−2^ s^−2^ irradiance (SL, 0.76 ± 0.06 day^−1^ growth rate at time of infection) or shifted to 15 μmol photons m^−2^ s^−2^ irradiance (LL, 0.091 ± 0.082 day^−1^ growth rate at time of infection). A,B. Viral production is shown for SL (filled triangles) and LL (open triangles) treatments as the fold change in virion abundance relative to abundance at T = 0 h to account for the different initial concentrations of total virions added to OlV1‐ vs. OlV7‐infected cultures (Table 1). C. The relative sensitivity of OlV1 (black triangles) and OlV7 (grey triangles) to host growth at reduced irradiance is shown as the ratio of viral production from LL and SL cultures at each time point after the release of progeny virions. A ratio of 1 indicates that an equal proportion of virions were produced under the different host growth conditions. Points show mean ± standard deviation of biological replicates (*n* = 3). Shaded areas indicate dark period in 14:10 h diel cycle.

Relative production of both OlV1 and OlV7 was sensitive to host growth physiology (Fig. 4A and B). We observed a significantly higher (two‐sample *t*‐test*, P* < 0.01) relative increase of OlV7 virions (log2FC = 5.6 ± 0.4 at 24.5 h) than OlV1 virions (4.0 ± 0.4) in SL (Fig. 3B). For both viruses, significantly fewer virions were produced in LL as compared to SL by 8.5 h after inoculation (Welch's two‐sample *t*‐tests, *P* < 0.02). By the end of the infection cycle, production of OlV7 virions decreased by a greater margin between SL and LL (48.6 ± 14.2 fold change in SL vs. 16.6 ± 1.2 in LL, Fig. 4B) than OlV1 production (16.1 ± 3.6 fold change in SL vs. 9.2 ± 1.1 in LL, Fig. 4A), although relative production of OlV7 was still higher than OlV1 (Supporting Information Fig. S2B). To test whether OlV7 is more sensitive to host growth physiology than OlV1, we calculated virus‐specific ratios of production between SL and LL conditions at time points following the initial release of progeny virions (8.5 h; Fig. 4C, using the abundance metrics shown in Fig. 4A and B). Pairwise comparisons show that the magnitude by which viral production is reduced under LL growth is consistently greater for OlV7 than OlV1 (i.e., OlV7 production is more sensitive), although the difference in means is only statistically significant at 8.5, 10.5 and 24.5 h (*P* < 0.05). These findings suggest that replication of OlV1 is more resistant to changes in *O. lucimarinus* growth rate than OlV7, providing further evidence for the functional diversity of these phylogenetically related marine viruses.

### *Infection phenotypes and physical interactions with host cells*

To gain insights into potential factors underlying the observed differences in infection by OlV1 and OlV7, we compared a series of virus‐focused measurements and calculations. OlV7 appeared more efficient at infecting host cells than OlV1 (Fig. 3D), resulting in a greater relative increase of viral progeny (Fig. 3B), despite a lower initial ratio of infectious virions per host cell (Table 1). Data from SYBR staining and flow cytometry were used to calculate percentages of infected host cells in each virus treatment (Fig. 3D) based on relative changes in SYBR‐based DNA content (i.e., reflecting replicated viral genomes) after accounting for cell cycle related changes in DNA content. OlV1 and OlV7 both showed a peak in proportion of infected cells between 6.5 and 10.5 h after addition of virus in SL, but OlV7 infected a significantly higher proportion of the host population overall (73% ± 4%) than OlV1 (36% ± 9%; Welch's two‐sample *t*‐test, *P* < 0.02). The mean fraction of OlV1‐ and OlV7‐infected cells was not significantly different (*P* > 0.05) at early (0.5–4.5 h) and late (18.5 and 24.5 h) time points, indicating that differences between the viruses were most pronounced during the middle stages of the infection process, whereas non‐infected controls were progressing through cell division.

OlV7 reached maximum disappearance from the medium by 6.5 h after addition of virus (log2FC = −0.43 ± 0.13; one‐sample *t*‐test for mu = 0, *P* < 0.05), suggestive of adsorption to host cells. For OlV1, significant changes were not observed over the same time interval (Fig. 3B). Due to its lower infectivity, OlV1 was added to host cultures at a higher virus: host ratio (SYBR‐determined) than OlV7 treatments (25 vs. 4.8‐5.5) to achieve a similar MOI (Table 1). Consequently, the variation (i.e., standard deviation) in SYBR‐determined OlV1 counts at early time points (2.5, 4.5 and 6.5 h) was 9–50 times that of OlV7 counts, and this higher background may have obscured detection of an adsorption signal in OlV1. Because some adsorption must have occurred to produce OlV1‐infected host cells, we applied an additional assay to estimate infection by OlV1. Fluorescently labelled probes designed here for hybridization of *O. lucimarinus* or OlV1 (Supporting Information Table S4) and applied to a single biological replicate of the SL and LL treatments across time points showed qualitatively that viruses were attached to host cells by 0.5 h (Supporting Information Fig. S3), when statistically significant disappearance of OlV1 virions was not yet detected by flow cytometry (Fig. 3B). In addition, these hybridizations qualitatively showed that lysed host cells were present by 4.5 h after inoculation (Supporting Information Fig. S3) before statistically significant increases in free virions (Fig. 3B, Supporting Information Fig. S2B) or decreases in host abundance (Fig. 3A, Supporting Information Fig. S2A). For both OlV1 and OlV7, the first significant increase in free‐virion abundance was observed 8.5 h after inoculation (*P* < 0.02). This demonstrated that OlV1 and OlV7 have similar latent periods (between 6.5 and 8.5 h), at least at the resolution of our study, confirming a previously published estimate of virion release by approximately 8 h post‐infection (Derelle *et al.,* 2015).

The approximate number of virions released from each lysed host cell (estimated burst size) varied substantially among biological replicates. We did not detect statistical differences between OlV1 (435 ± 251 virions per cell) and OlV7 (682 ± 408 virions per cell) in SL (Table 1). Collectively, our results suggest that although OlV1 and OlV7 share similarities in their latent periods and burst sizes, OlV7 may more efficiently adsorb to *O. lucimarinus* host cells than OlV1, facilitating infection of a higher proportion of the host population (Fig. 3D) and resulting in a greater yield of viral progeny (Fig. 3C).

### *Dissolved organic matter from lysed hosts*

**Figure 5:**
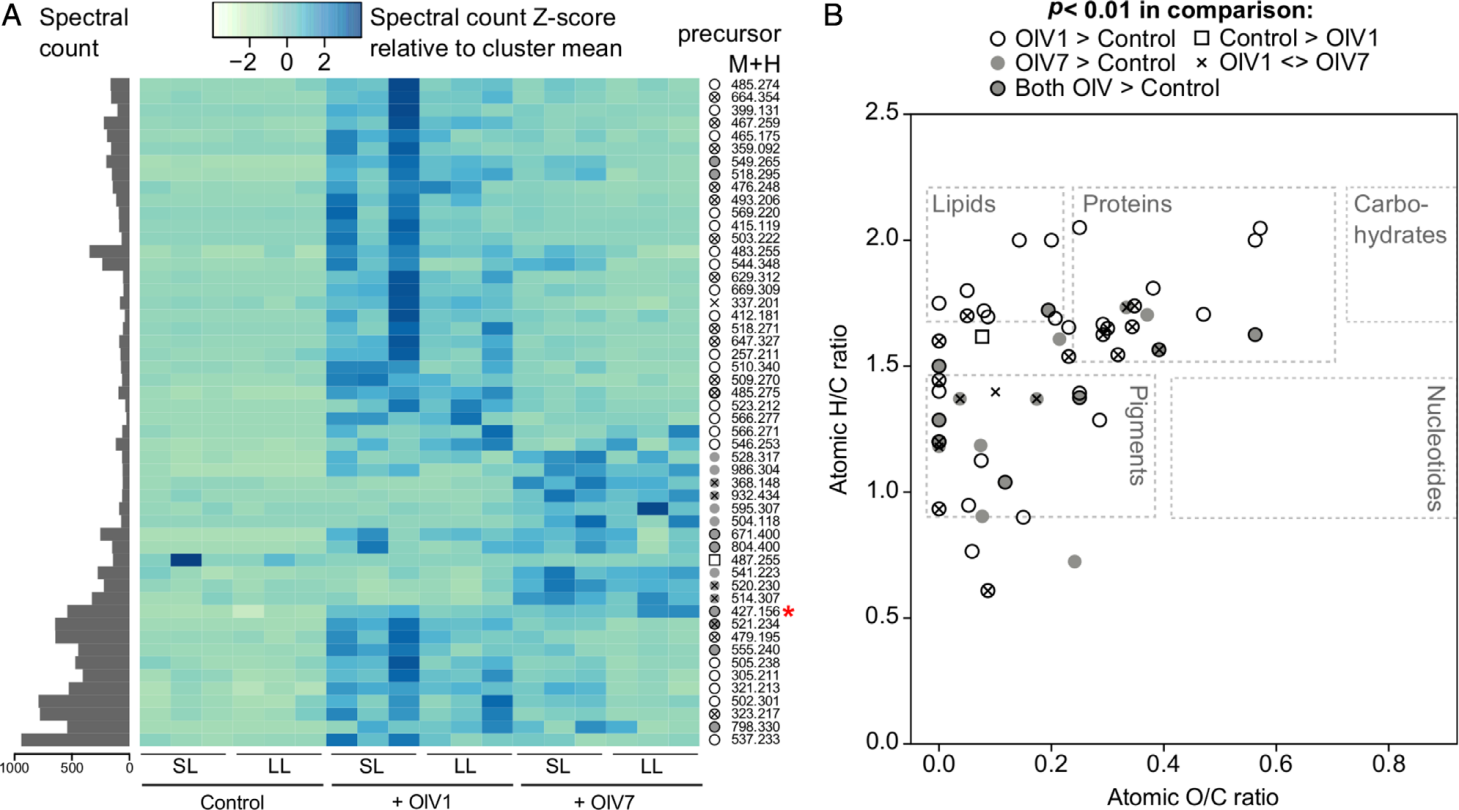
Differential chemical composition of dissolved organic matter resulting from virally infected host cultures (‘+OlV1’ and ‘+OlV7’) as compared to non‐infected control cultures acclimated to 105–115 (SL) and 15 (LL) μmol photons m^−2^ s^−2^ irradiance. A. Heatmap of abundance of spectral clusters that were significantly (*P* < 0.01) differentially abundant between pairwise comparisons of virus treatment groups (represented by different symbols). Only one spectral cluster was also found to have significant differential abundance between SL and LL conditions (red asterisk). The heatmap is coloured based on spectral counts in a given sample relative to the cluster mean across samples. Total spectral count across samples is shown for each significant cluster on the left (grey bars). On the right side, compound mass is expressed as monoisotopic M + H mass for each spectral cluster. B. van Krevelen diagram (i.e., elemental ratio plot) for best‐fit molecular formulas (lowest mass error from observed cluster molecular weight) of statistically significant spectral clusters. Clusters are represented by different shapes indicating the direction of significant differences between virus treatments. The approximate composition regions for some major classes of biochemicals are indicated.

One of the viral impacts frequently discussed in the literature is that lysis and release of host material will likely modify the chemical environment for the natural microbial community (Fuhrman, 1999; Wilhelm and Suttle, 1999; Weitz *et al.,* 2015), although this has not been explicitly tested for environmentally relevant marine eukaryotes. Therefore, here dissolved organic matter (DOM) resulting from viral lysis was characterized to determine the extent to which viral infection modifies the chemical nature of compounds released from host cells and to evaluate whether observed differences in OlV1 and OlV7 infection dynamics translated into differential impacts on the organic matter available in the surrounding environment. DOM released from a combination of actively infected (i.e., exudates) and lysed *O. lucimarinus* host cells sampled at 24.5 h post‐infection showed a number of chemical signatures distinct from DOM from uninfected cells, and some of those signatures were specific to either OlV1 or OlV7 infection (Fig. 5A). Analysis by high‐resolution LC–MS/MS of DOM revealed 52 spectral clusters (out of a total of 4969 comparisons) with significant abundance differences (*P* < 0.01) between the non‐infected controls and either (or both) infected treatment(s), when SL and LL conditions were pooled. Fifty‐one of the 52 spectral clusters were more abundant in infected samples. Eight clusters were more abundant in both infected treatments compared to the non‐infected controls, whereas 34 were specific to OlV1‐treatments and 9 to OlV7‐treatments relative to controls. Fewer distinct clusters were detected as significantly enriched in OlV7 treatments (17 clusters), where 77%–92% of cells had lysed, than in OlV1 treatments (42 clusters), where lysis rates were 27%–51%. When the abundances of these 52 ‘infection‐associated’ spectral clusters were compared between SL and LL conditions within each viral treatment (i.e., OlV1 SL vs. LL, OlV7 SL vs. LL and non‐infected controls SL vs. LL), only one cluster showed significant differential abundance (higher in OlV7 LL than SL, Fig. 5A). Statistical significance of other spectral clusters that appear at least qualitatively differentially abundant between SL and LL conditions may have been obscured by heterogeneity among biological replicates. Best‐fit molecular formulae assigned to the cluster precursor masses suggest that many of the DOM compounds characteristic of viral lysis have pigment‐, protein‐ and lipid‐like compositions (Fig. 5B), although these compounds could not be more specifically identified by comparison with current metabolite mass spectral databases or proteomes predicted from the virus and host genomes. Overall, these results suggest that similar biomolecules were enriched in the organic matter released from infection by either virus, but that specific molecular signatures from lysis by OlV1 or OlV7 could be discerned with high‐resolution analyses.

## Discussion

### *Closely related viruses by conventional metrics manifest different infection strategies*

Here, we have compared the infection dynamics of two of the closest known viruses in culture that infect the same eukaryotic marine phytoplankton species. Of the seven viruses that have been isolated against *O. lucimarinus*, OlV1 and OlV7 branch closest together among the currently sequenced suite of *Ostreococcus* viruses, adjacent to each other in our (Fig. 1A) and other (Derelle *et al.,* 2015) phylogenomic analyses. Our re‐purification to pure viruses followed by imaging, resequencing and genome assembly, established that these viruses maintained prior reported morphological characteristics despite time in culture, and had largely co‐linear genomes, as reported when they were initially isolated (Derelle *et al.,* 2015). Moreover, based on one of the most commonly used molecular markers for investigating phycodnavirus diversity, the DNA polymerase gene (PolB) (Chen and Suttle, 1995; Chen *et al.,* 1996; Larsen *et al.,* 2008; Clerissi *et al.,* 2014), their aa identity is 99.5% and 98.0% at the nucleotide level, indicating that most variation is in the third codon and hence has little impact at the protein level.

Our experimental conditions were comparable for tests on the two *O. lucimarinus* viruses, optimized through preliminary studies on the particular eukaryote under investigation as recommended in several recent reviews (Massana and Logares, 2013; Worden *et al.,* 2015; Caron *et al.,* 2016). This enabled us to establish key differences in the ecological dynamics of host and virus populations using high‐resolution sampling, intentional consideration of host physiology before and during infection and assessment of virus–host interactions in variable host growth conditions. OlV1 and OlV7 showed distinct infection phenotypes that had different consequences for host population dynamics and, consequently, would differentially impact host ecology in the field as well as associated aspects of dissolved nutrient cycling. OlV7 has a more virulent lytic infection than OlV1, which was characterized by early arrest of the cell cycle and rapid lysis of host cells, resulting in a near‐50‐fold production of progeny virions. By comparison, OlV1 appears to have commandeered host cellular machinery differently, such that host cells continued through ‘normal’ cell cycle phases early during the infection, infected significantly fewer cells in the host populations and produced only a 16‐fold increase in progeny virions by experiment termination.

### *Potential genetic basis of different infection phenotypes*

Although OlV1 and OlV7 are highly related based on comparison of core prasinovirus genes, they nevertheless each contain sets of non‐orthologous genes, comprising 12% and 9% of their predicted proteomes, respectively. Most of these (as well as most of the orthologous genes) lack recognizable functional domains (Supporting Information Table S3); thus, the functional characterization of viral genes is a fruitful area of future research. However, there were aspects of the unique gene sets worth noting. OlV7 generally showed enrichment of functions involved in gene expression, such as expansion of the Fe(II)/2‐oxoglutarate‐dependent oxygenase protein family, which encompasses diverse functions affecting multiple steps in transcriptional and translational processes, as well as biosynthesis and degradation of cellular metabolites (Herr and Hausinger, 2018). Several cyanophage genomes also encode multiple copies of 2OG‐Fe(II) oxygenase superfamily proteins, presumed to function in DNA repair (Weigele *et al.,* 2007; Sullivan *et al.,* 2010). An ortholog of a putative MYM‐type Zinc finger domain that functions as a transcriptional trans‐activator in *Vaccinia* virus (Keck *et al.,* 1993) was found in OlV7 and all prasinoviruses compared here, except OlV1 (Supporting Information Table S3). OlV7 also encodes a putative ketopantoate hydroxymethyltransferase, the first enzyme in pantothenate biosynthesis, which is a necessary precursor to coenzyme A. The protein catalyses a rate‐limiting step in the synthesis of vitamin B5 in *E. coli* (Teller *et al.,* 1976) and is localized to the mitochondria in higher plant cells (Ottenhof *et al.,* 2004). Orthologous proteins were identified in three of OlV Type II subgroup viruses (OlV2, OlV5 and OlV6) and OtV‐2 (Weynberg *et al.,* 2011), which infects the more oligotrophic‐optimized *Ostreococcus* Clade OII (Demir‐Hilton *et al.,* 2011). Although the role of this putative ketopantoate hydroxymethyltransferase during infection is unknown, it could augment essential enzyme cofactors for a variety of biosynthetic pathways in the host during infection (Kleinkauf, 2000).

In contrast to OlV7, OlV1‐specific genes appear to be enriched in biological functions related to host interactions and metabolism, including fatty acid and aa biosynthesis (Supporting Information Table S3). For example, the OlV1 putative rhamnose synthetase may be involved in making substrates for glycosyltransferase, which are postulated to be involved in post‐translational modification of capsid proteins in chloroviruses (Parakkottil Chothi *et al.,* 2010; and references therein). Orthologues of the putative rhamnose synthetase were identified only in OtV5 and OmV1 (Supporting Information Table S3). Weynberg *et al*. (2017) hypothesized that glycan‐mediated virus–host interactions may be one of the key points of host resistance to infection. The presence of this putative synthetase in OlV1 and plausible incorporation of rhamnose glycoconjugates in the OlV1 virion capsid allude to possible differences in host specificity between OlV1 and OlV7. Indeed, the low virulence of OlV1 on *O. lucimarinus* CCMP2972 as compared to OlV7 could suggest that distinct OlV1 capsid glycoproteins may not efficiently recognize and attach to this host's cell surface. Although both OlV1 and OlV7 appear to be restricted to *Ostreococcus* Clade OI (A) hosts (Derelle *et al.,* 2015), OlV1 may exhibit more virulent infection of other OI strains. Interestingly, CCMP2972 was isolated from Pacific coastal waters near San Diego, CA, USA (Worden *et al.,* 2004) and OlV7 from the central coast of CA, USA, whereas OlV1 was isolated from coastal Mediterranean waters (Moreau *et al.,* 2010; Derelle *et al.,* 2015). Notably, OlV1 and all other genome sequenced OlVs, except OlV7, encode a putative high affinity phosphate transporter (PHO4) (Derelle *et al.,* 2015) that is present in many other prasinophytes and their viruses (Monier *et al.,* 2012). Because phosphate was not limiting in our studies, we do not expect that the lack of PHO4 in OlV7 contributed to the observed differences in infection phenotype. However, virally encoded PHO4 is hypothesized to enhance phosphate uptake during infection under phosphorus‐starved host growth (Monier *et al.,* 2012), potentially alleviating limitation of an essential nutrient for replication of viral genomes, as has been observed for the PstS phosphate transport system expressed by cyanophages (Zeng and Chisholm, 2012). Thus, the virulence of OlV7 infection may be reduced as compared to OlV1 in environments where the host otherwise cannot successfully compete for scarce phosphate supplies. These suites of unique genes, although relatively few, are important to consider when extrapolating phylogenetic inferences, which are by necessity based on shared components, to functional significance of diversity.

### *Functional comparison at various stages of infection*

The molecular determinants of infection (e.g., identity of cell surface receptors targeted by viruses, host defence mechanisms) remain unknown for many virus–host pairs, challenging extrapolation of genetic data to predictions for virus–host interactions. However, further insight into the key biological differences between OlV1 and OlV7 infections can be gleaned by considering individual steps within the infection process. The rate at which a virion encounters a susceptible host cell critically limits the propagation rate and outcome of viral infection (Murray and Jackson, 1992; Mann, 2003; Brown and Bidle, 2014). We controlled the contact rate in our experimental design by standardizing culture volumes, host cell densities and the ratios of infectious virions per host cell (i.e., MOI) which was tested before and after each experiment. The MOI of infected cultures in our experiment was higher for OlV1 than OlV7 (3.4 vs. 1.5‐1.7, Table 1), enabling a higher encounter rate between host cells and infective virions and potentially a higher proportion of infected cells in these treatments. However, we observed the opposite trend that OlV7, despite its lower MOI, clearly infected more cells in host populations than OlV1 (Fig. 3). Therefore, our observations likely underestimate the differences in infection efficiency between OlV1 and OlV7, such that OlV7 would appear to be even more virulent if assessed at an equivalent MOI.

Assuming that the disappearance of viral particles early in our experiments was correlated with adsorption to host cells, it is plausible that OlV1 and OlV7 differ in their adsorption efficiency, at least to the host strain tested. The exact mechanisms of adsorption and host cell membrane fusion are not known for these viruses. Prasinoviruses encode eight major capsid protein (MCP) genes (Weynberg *et al.,* 2017), and OlV1 and OlV7 vary in as few as zero and as many as eight aa positions across these genes (mean = 3, median = 2), a factor hypothesized to affect host specificity of dinoflagellate RNA viruses (Nagasaki *et al.,* 2005). These subtle differences in capsid proteins may indicate differences in host strain specificity between OlV1 and OlV7, such that OlV1's specific complement of capsid proteins is less effective at adsorbing to *O. lucimarinus* CCMP2972 cell surface receptors than OlV7. As discussed above, these differences could also be connected to divergence between Clade OI strains from the Pacific Ocean, such as CCMP2972 (Worden *et al.,* 2004), and those from other ocean regions such as the Mediterranean Sea from which OlV1 was isolated.

Once inside the host cell, successful infection depends on the virus' ability to evade and/or inactivate host defences, as well as take over host transcription and translation machinery. The genomes of both viruses encode methyltransferases, presumably involved in protecting DNA from host enzymes (Zhang *et al.,* 1992; Agarkova *et al.,* 2006), as well as suites of DNA replication (e.g., DNA polymerase, ligase, topoisomerase) and transcription enzymes (e.g., transcription factor‐like elements, mRNA‐capping enzymes). Although not quantified here, transcriptional and translational responses, of orthologous genes or unique genes, have the potential to influence differences in infection dynamics. We hypothesize that in addition to likely differential adsorption efficiencies, OlV7's gene repertoire facilitates more efficient takeover of host transcription and/or translation machinery, leading to the observed differences in the proportion of infected cells in the host population between OlV1 and OlV7 (difference > 30% over multiple time points, Fig. 3D). The host may also exhibit differential resistance to infection by these viruses. The small outlier chromosome in *O. tauri* has been proposed to play a role in viral resistance (Yau *et al.,* 2016). The corresponding smallest chromosome(s) in *O. lucimarinus*, as well as genome‐sequenced *Micromonas* species, have overarching similarities to that of *O. tauri* both in terms of differentiated GC content and gene structure from other chromosomes (Worden *et al.,* 2009; Moreau *et al.,* 2012). Characterization of gene expression during infection should reveal whether *O. lucimarinus* transcriptional responses differ under infection by OlV1 versus OlV7, and the extent to which transcriptional patterns may apply more broadly to algal virus–host pairs.

### *Viral traits and potential trade‐offs*

Several other factors must also be considered while interpreting the dramatic differences in infection kinetics observed between OlV1 and OlV7. Although the replicative cycle of OlV1 was less virulent than OlV7 regardless of host growth rate, OlV1 production was more robust to light‐limited (i.e., energy‐limited) host growth than OlV7 (Fig. 4C). One caveat of our study is that viral production was measured only as the change in total virion abundance, but quantification of infectious virions would provide additional insight into the influence of host growth rate on infection. The resilience of OlV1 infection to host growth physiology may suggest a potential fitness trade‐off for these eukaryotic viruses between infection efficiency and plasticity (i.e., flexibility to accommodate different host growth conditions; as proposed for *E. coli* phages, Choua and Bonachela, 2019). Our results imply that OlV7's ability to efficiently replicate under healthy host growth conditions may come at the expense of being able to cope with variable host growth, whereas a consistently less virulent/productive infection by OlV1 may be compensated for by a more limited dependence of replication on host physiology.

Few studies have explicitly evaluated the influence of host growth rate associated factors on the life cycles of eukaryotic viruses (discussed in Bachy *et al.,* 2018), although metabolic rate and abundance and activity of ribosomes have been shown to affect phage production rates (Middelboe, 2000; You *et al.,* 2002; Nabergoj *et al.,* 2018). For a prasinovirus infecting *Micromonas* sp. RCC829 (Clade B.E.3), latent period decreased and viral production rate increased significantly with increasing host growth rate over a range of temperatures from 9.5°C to 25°C (Demory *et al.,* 2017). However, the sensitivity of viral production to host physiology may be dependent on the specific host or virus and the factor used to modulate host growth rate. *Micromonas* species exhibit variably lower growth rates when cells are grown in phosphate‐ and nitrate‐deplete conditions (Maat and Brussaard, 2016; Bachy *et al.,* 2018; Guo *et al.,* 2018) or in reduced light (i.e., reduced energy availability) (Baudoux and Brussaard, 2008; Maat *et al.,* 2016; Piedade *et al.,* 2018), than in replete conditions. Although interpretations of these experiments vary, in those examining viral infection, the number of viral particles released by host cells after lysis appears to be lower in host limiting growth conditions. Collectively, these studies suggest an apparent dependence of viral production on host growth rate (or growth rate associated factors) that is to some degree independent of the rate‐limiting growth factor. However, our results are the first to show how these viral production‐related terms vary not just under two different host growth states, but also differ between two viruses of the same algal species under identical growth conditions.

Recent theoretical and empirical studies have identified several key traits implicated in life history trade‐offs for *E. coli* phages (De Paepe and Taddei, 2006; Bonachela and Levin, 2014; Keen, 2014), aquatic (freshwater and marine) cyanobacterial and microalgal phytoplankton viruses (Edwards and Steward, 2018), or marine viruses generally (Record *et al.,* 2016), including burst size and latent period (together representative of reproduction rate), genome size, capsid size/morphology, adsorption rate, virion stability and host range. The seven available genome sequenced OlVs, including OlV1 and OlV7, were isolated against *O. lucimarinus* CCMP2972, the genome sequenced representative of *Ostreococcus* Clade OI/A (Worden *et al.,* 2004; Palenik *et al.,* 2007). Notably, as discussed above, OlV7 was isolated from eastern North Pacific coastal waters that have high connectivity to the isolation site of *O. lucimarinus* CCMP2972, which is also in the eastern North Pacific, whereas the other OlVs come from environments ranging from the coast of southern Chile in the eastern South Pacific to the western English Channel near the coast of France. The OlVs do not appear to infect characterized members of other *Ostreococcus* clades, but OlV1 and OlV7, as well as some other OlVs, do differ in their ability to lyse isolates from different parts of the world that may belong to Clade A (Derelle *et al.,* 2015). To date, no studies have tested these viruses against other *bona fide* Clade A isolates with known 18S rRNA gene sequences. Distinctive clade specificities were observed for populations of viral isolates infecting *Micromonas*, with a few exceptions (Baudoux *et al.,* 2015). Thus, subtle differences in host range, suitability or virus–host coevolution could have played a role in the differences in our experimental results and would influence the effects of these viruses in natural populations.

Here, although virulence was clearly higher for OlV7 under both host growth rates tested, differences between OlV1 and OlV7 infection kinetics (burst size and latent period) only became apparent under reduced host growth rate (Table 1). We speculate that virion stability was not a major differentiating factor on the timescale of this study. Although prasinoviruses infecting different clades of the genus *Micromonas* exhibited variable decay rates across a range of temperatures (Demory *et al.,* 2017) and dsDNA viruses infecting another class of eukaryotic algae decayed more quickly in light vs. dark conditions (Tomaru *et al.,* 2005), loss of infectivity in both studies occurred over weeks. Furthermore, the differences in burst size and latent period were most prominent in LL (i.e., reduced light) where irradiance‐driven virion degradation would be expected to be lower than in SL. Within the context of Phycodnaviruses broadly, which range in genome sizes between 100 and >550 kb (Wilson *et al.,* 2009), OlV1 and OlV7 have relatively similarly sized genomes, with OlV1 being just 12 kb larger. We hypothesize that this small difference in genome size may contribute to observed higher relative production of OlV7 over the time frame of our experiment (Fig. 3B). In dsDNA T4 phages, the direct energetic cost of genome replication is calculated to outweigh translation costs at capsid diameters exceeding 80 nm (Mahmoudabadi *et al.,* 2017). Using their model to calculate the direct energetic cost of synthesizing the OlV genomes (see Supporting Information), we estimated that 300,000 additional ATP‐equivalents per OlV1 genome are needed for replication alone, exclusive of transcription and translation costs. This difference in energetic cost may be sufficient to allow faster OlV7 replication when host machinery is primed under ideal growth conditions. As noted above, several OlV1‐specific genes may help compensate host metabolism to some extent. Although empirical evidence is needed to support these hypotheses, identification and testing of potential trade‐offs is a key step toward accurately parameterizing ecological models.

### *Relevance to ecosystem ecology and marine biogeochemistry*

Our results indicate that in nature, infection of *O. lucimarinus* by either OlV1 or OlV7 would differentially impact host population dynamics. Considering that picoeukaryotes such as *Ostreococcus* can be major contributors to primary production (Li, 1994; Fouilland *et al.,* 2004; Worden *et al.,* 2004), the results observed for host dynamics can be expected to also differentially impact the productivity of the community and inter‐species interactions (e.g., competition, predation). In addition, analysis of the DOM collected at the end of the infection experiment showed that there were some chemical signatures specific to OlV1 or OlV7 infection, but more clearly demonstrated that organic matter released from viral infection is compositionally distinct from that of non‐infected controls (Fig. 5A). Only one spectral cluster was enriched in the non‐infected controls, whereas all other significantly differentially abundant clusters were more abundant in viral lysates. It should be noted that samples for DOM analyses were collected more than 16 h after lysis began (see Fig. 3), but before complete lysis of host cells, so that DOM composition likely represents a mixture of exudates and lysis products. Because infection by OlV7 was more virulent and more cells in the host populations had lysed at the time of sample collection, it is possible that the two viral treatments differed in the degree of post‐lysis degradation of organic matter, which could have influenced the abundance and/or chemical diversity of the higher‐molecular‐weight compounds best recovered by our extraction methods. Thus, our endpoint data does not address whether DOM compound abundance and/or diversity can be connected to the proportion of lysed host cells; however, experiments with time‐resolved DOM analysis could address this possibility.

Predicted molecular formulae indicated that infection‐specific spectral clusters were enriched in compounds that have pigment‐, protein‐ and lipid‐like compositions (Fig. 5B). This result is in contrast to virally released organic matter from the cyanobacterium *Synechococcus* WH7803, which was found to have many more unsaturated, polyphenolic‐like compounds (Ma *et al.,* 2018). In our culture‐based study, we were unable to directly link putative peptides detected in the DOM to their protein sources, although both virus and host have completely sequenced genomes and predicted proteomes. Likewise, when a similar high‐resolution analysis was applied to Pacific coastal seawater collected near San Diego, CA, only 0.5%–1% of detected DOM compounds could be annotated using available datasets (Petras *et al.,* 2017). Further development of DOM analysis as a tool to assess environmental virus–host interactions will depend on continued expansion of metabolite libraries and analytical techniques. Even so, our results shed light on the interplay between viral infection and the composition of resulting DOM in an environmentally relevant marine picoeukaryote. These findings support that infected cells (intact and lysed) are biochemically distinct from their non‐infected counterparts (Ankrah *et al.,* 2014; Ma *et al.,* 2018), and that viral infection modifies the biochemistry of the environment. In natural communities, viral lysis is predicted to impact multiple ecosystem‐level processes, including remineralization of essential nutrients, transfer of organic matter to higher trophic levels and primary (and bacterial) production rates (Fuhrman, 1999; Weitz *et al.,* 2015). However, the direction and magnitude of these effects depend on the specific composition and bioavailability of cellular compounds released by viral lysis, which likely vary across diverse phytoplankton virus–host pairs but remain poorly characterized.

### *Conclusions*

Our studies provide evidence for differential and ecologically relevant consequences of infection by the most closely related prasinoviruses, with 98.0% PolB nucleotide identity, known to infect the prominent marine picophytoplankton species *O. lucimarinus*. In addition to distinct impacts on host physiology, population dynamics and specific chemical composition of viral lysate, our comparative analysis revealed differential dependence of these viruses on host growth, suggesting an intriguing potential fitness trade‐off that merits further investigation. The observations presented here underscore the significant biological insights that can be gained from evaluation of multiple virus–host interactions over a range of host growth rates, better reflecting the range that is encountered in nature. These observations also provide valuable context for interpretation of expanding catalogues of environmental viral diversity. Characterization of DOM resulting from viral lysis provides a critical link to specific impacts on biogeochemical cycling and importantly shows differences in DOM derived from virally lysed cultures versus uninfected cells. The key functional differences—amounting to traits—and possible trade‐offs associated with related viruses infecting the same host species represents an important consideration for ocean ecosystem models.

## Experimental procedures

### *Algal host growth conditions*

Axenic *Ostreococcus lucimarinus* CCMP2972 (CCE9901) (Worden *et al.,* 2004) cells were grown in L1 media (pH = 8.3, salinity = 35 ppm; Guillard and Hargraves, 1993) made from a natural seawater base (collected from Station 67‐135 at 33.953°N, −128.048°E in October 2011) amended with 0.01 μM H_2_SeO_3_ (as per Worden *et al.,* 2004). Cultures were maintained at 18°C under a 14:10 h light:dark cycle with a fluorescent light irradiance of 105–115 μmol photons m^−2^ s^−1^ (termed here ‘SL’ for Standard Light) for 9 days (>7 generations) in exponential growth by semi‐continuous batch culturing with daily transfer or dilution to 5.2 ± 0.5 × 10^6^ cells mL^−1^. An Accuri C6 flow cytometer (BD Biosciences) was used to monitor growth of live cells daily. Axenicity was assessed by 4,6‐diamidino‐2‐phenylindole (DAPI) staining (Porter and Feig, 1980) followed by visual inspection using epifluorescence microscopy. In addition, culture samples were inoculated into an organic rich test medium and checked for bacterial growth after incubation at room temperature in the dark for up to 1 week. Cultures were not diluted the day before experiment setup to increase biomass but were still in mid‐exponential growth.

### *Virus preparation*

Lytic viruses OlV1 and OlV7 were used for all infection experiments (Derelle *et al.,* 2015). Two rounds of serial dilution to extinction were then performed to ensure purity of each virus sample and confirmed through re‐sequencing the genomes (described below). Fresh batches of viral lysate were prepared immediately before experiments from a master stock of each virus to ensure a high proportion of infectious virions and to reduce the probability of introducing genetic mutations through serial passage (Zimmerman, 2016b). For this, exponentially growing CCMP2972 (growth rate = 0.65 ± 0.06 day^−1^ over 6 days, target host cell density at time of infection = 5 × 10^6^ cells mL^−1^) were infected by adding 1% (v/v) of virus master stock. Infected cultures were swirled and allowed to lyse under normal growth conditions until clear (5 days). Remaining host cells were removed by gentle vacuum filtration through a sterile Nalgene Rapid‐Flow 0.45 μm PES membrane filter. Viruses were concentrated using VivaSpin20 (Sartorius) 100,000 MWCO PES centrifugal filtration units. Each VivaSpin20 unit was washed twice with 0.02‐μm‐filtered (Whatman Anotop Plus) 1X TE buffer (pH 8.0) by gentle vortexing to dislodge additional virus particles.

The concentration of total virions was determined by analytical flow cytometry using a BD Influx cell sorter (BD Biosciences) equipped with a 488‐nm argon laser and flow meter, following procedures adapted from (Brussaard, 2004; Brussaard *et al.,* 2010). Samples of each concentrated viral lysate were fixed with glutaraldehyde (EM grade, 30 min at 4°C, 0.25% final concentration) and flash frozen. Immediately after thawing, each sample was diluted 1:10,000–1:20,000 in 0.02‐μm‐filtered TE buffer (pH 8.0) and stained in the dark with SYBR Green I nucleic acid dye (0.5× final concentration; Molecular Probes, Inc.) for 15 min at room temperature. Virion populations were resolved by green fluorescence (520 ± 35 nm filter, trigger) and Forward Angle Light Scatter (FALS) (Fig. 2B). Events were collected for 2–4 min, and the volume analysed was determined by weight measurements. Fluorescent polystyrene microspheres were added to each sample for reference (0.5 μm Green and 0.75 μm Yellow‐Green; Polysciences). Samples of TE buffer without virus were treated as described and used to correct virion counts for background noise.

The proportion of infectious viruses (those capable of entering host cells and producing a complete infection and lysis of host cells) in each of the concentrated viral lysates was estimated by an MPN assay (Taylor, 1962; Zimmerman, 2016a). Briefly, 50 μl serially diluted (10^−3^ to 10^−10^) fresh viral concentrate was added to 150 μl exponentially growing host cells (growth rate = 0.64 ± 0.18 day^−1^ over 4 days, target host cell density = 5 × 10^6^ cells mL^−1^) in triplicate 96‐well microtiter plates (24 replicate wells for each dilution) and incubated at normal growth conditions for 12 days. Cell lysis was assessed intermittently over 2 weeks visually and by measuring optical density on a plate reader (Molecular Devices SpectraMax 340PC) at 750 nm absorbance. The MPN of infectious viruses was estimated from the proportion of infection‐positive (i.e., lysed) wells using the MPN_ver4.xls Excel spreadsheet from (Jarvis *et al.,* 2010). Infectivity was then calculated by comparing the MPN‐estimated abundance of infectious viruses to the abundance of total virions determined by SYBR staining.

### *Viral genome analysis*

Viral DNA was isolated using a modified CTAB extraction procedure (Winnepenninckx *et al.,* 1993) from concentrated viral lysate collected on a 0.1 μm Supor PES membrane filter (Pall Corp.). Libraries were prepped using the NexteraXT DNA Library Preparation Kit (Illumina) according to the manufacturer's instructions. Libraries were sequenced using a NextSeq series instrument with 150 bp single‐end reads (Illumina).

Viral genome assemblies were assembled using SPAdes (v3.6.1), with the ‘single‐cell’ option activated and all other parameters set to their default value (Nurk *et al.,* 2013). Circular contigs were detected for each, indicating completed genomes. Genes were predicted with MetaGeneAnnotator (Noguchi *et al.,* 2008). All translated aa sequences were used in a blastp search (Altschul *et al.,* 1990) of the NCBI viral protein database (RefSeqVirus) for taxonomic affiliation (*E*‐value ≤10^−3^ and bit score ≥50). Functional annotations were derived from the PFAM database of protein domains (Punta *et al.,* 2012) using Hmmsearch (*E*‐value ≤10^−5^; Eddy, 2011).

A maximum likelihood phylogenetic tree was reconstructed based on alignment of 22 concatenated orthologous ‘core green algal virus’ genes (as in Derelle *et al.,* 2015). Amino acid sequences from available prasinovirus genomes as well as from several chloroviruses were aligned using M‐Coffee (Notredame *et al.,* 2000; Wallace *et al.,* 2006) and manually curated. The best maximum‐likelihood phylogenetic tree was constructed using the RAxML v7 (Stamatakis, 2006) hill‐climbing algorithm and PROT+CAT substitution model with 100 replicate reconstructions to compute bootstrap support.

ProgressiveMAUVE (Darling *et al.,* 2010) was used to align the whole genomes of OlV1 and OlV7 at the nucleotide level to confirm synteny. Orthologous protein coding genes and average aa identity were determined using the aai.rb script in the Enveomics collection (Konstantinidis and Tiedje, 2005; Rodriguez‐R and Konstantinidis, 2016) set to 20% minimum alignment identity and 50% minimum alignment length of the shorter sequence (thus, non‐orthologous genes were defined here as aa identity <20% and/or coverage <50%). Additional orthologues were identified using OrthoFinder default settings (Emms and Kelly, 2015), run with all the predicted protein sets from green algal viruses (prasinoviruses and closely related chloroviruses) downloaded from NCBI.

The re‐sequenced and annotated genomes were submitted to GenBank under the accession numbers MK514405 (OlV1) and MK514406 (OlV7).

### *Morphological analysis of viruses*

Viral lysates (10 μl) were deposited onto formvar‐coated 200 mesh copper TEM grids (Ted Pella) and incubated for 15 min at room temperature. The remaining volume was removed, and an additional 10 μl lysate were deposited and incubated for 15 min. Grids were washed with distilled water twice and negatively stained with 10 μl 2% uranyl acetate for 15 s. Samples were imaged on a FEI Tecnai G2 Spirit TEM at an acceleration voltage of 80 kV. Viral capsid diameters were measured using ImageJ 1.50i software (Abràmofff *et al.,* 2004).

### *Viral infection experiment*

CCMP2972 host cultures were pooled and concentrated by centrifugation (twice for 30 min at 10,000*g* and 20°C; washed in between with sterile media). Concentrated cells were used to establish experimental flasks at two irradiance levels: SL (105–115 μmol photons m^−2^ s^−2^, equivalent to standard host growth conditions) and LL (15 μmol photons m^−2^ s^−2^). All other growth conditions were as stated above. The SL condition was inoculated at 6.5 ± 0.8 × 10^5^ cells mL^−1^, whereas the LL condition was inoculated at 1.3 ± 0.2 × 10^6^ cells mL^−1^ to account for anticipated differences in growth rate before infection. Cultures were allowed 1 day to recover from centrifugation before shading was added to reduce irradiance for the LL treatment. Growth was monitored daily by flow cytometry. Cultures were acclimated to experimental conditions for 3 days (in mid‐exponential growth) without transfer or dilution leading up to viral infection. Preliminary experiments showed that this timeframe was sufficient to observe a reproducible response in host physiology to the LL condition, evidenced by a substantial difference in growth rate from the SL condition at the time viruses or buffer were added, but without additional reduction in growth rate thereafter (Supporting Information Fig. S4).

After acclimation to the experimental set up, measured host cell densities (collected at dawn, 3.5 h before addition of virus) were used to calculate the volumes of viral lysate needed to achieve a target multiplicity of infection (MOI, ratio of infectious viruses to host cells) of 3. Average cell density across both irradiance conditions was 3.2 ± 0.6 × 10^6^ cells mL^−1^, and the growth rate of the SL condition on the day of the experiment was 0.76 ± 0.06 day^−1^ whereas the LL condition was 0.091 ± 0.082 day^−1^ (calculated over the 24 h before addition of virus). To provide an equal number of infectious particles, we used preliminary MPN assay experiments to guide the addition of viral lysate. OlV1 was estimated to be 14% infective, and OlV7 was estimated to be 65% infective, resulting in a higher ratio of total virions added to host cells in OlV1 treatments (25) as compared to OlV7 treatments (5–6 virions per host cell). Viral inoculum represented 1.0% ± 0.1% culture volume. MPN assays were repeated on the inoculum used in the experiment to confirm infectivity and MOI at the time of infection. Triplicate non‐infected control cultures for each irradiance condition were inoculated with 0.02‐μm‐filtered TE buffer instead of viral lysate.

After addition of viruses or buffer, all flasks were mixed by hand and initial (*T* = 30 min) samples were collected. Culture flasks were repeat‐sampled every 2 h for the first 14 h, then at 18 and 24 h following the addition of viruses. Flow cytometry samples were preserved with glutaraldehyde as described above and stored at −80°C until analysis. Samples for viralFISH were collected every 4 hours, fixed with 2% paraformaldehyde (v/v, final concentration) at room temperature for 1 h before flash freezing in liquid nitrogen. End‐point samples for dissolved organic matter (DOM) analysis were collected from all flasks by filtration through pre‐combusted (3 h, 450°C) 25 mm glass fibre filters (0.3‐μm nominal pore size, Sterlitech Corp.). Filtrates were collected in acid‐cleaned 50 ml conical tubes and stored at −20°C until further processing.

### *FCM sample processing*

Samples for virion and host cell counts were quantified by flow cytometry. Briefly, glutaraldehyde‐preserved samples were individually thawed and immediately diluted 1:5 to 1:25 in 0.2‐μm‐filtered (Whatman Puradisc 25 PES) 1× PBS (pH 7.4) to resolve host cell populations by FALS (trigger) and red chlorophyll autofluorescence (692 ± 40 nm filter) (Fig. 2A). Concurrently, samples were also diluted 1:100 to 1:20,000 in 0.02‐μm‐filtered TE buffer, stained with SYBR Green I and analysed as described above for virion counts and host cell cycle analysis. The frequencies of host cells in G1 phase at each time point were delineated from non‐infected control cultures (after gating the host cell population by FALS and chlorophyll autofluorescence) as the major peak in SYBR (i.e., DNA) fluorescence histograms, which was exactly half the fluorescence of a secondary peak (Supporting Information Fig. S5). Host cells with SYBR fluorescence above this threshold were considered to be infected with replicating viruses and/or in S, G2 or M phases (i.e., DNA content greater than one host genome). Flow cytometry data were analysed using WinList (Verity Software House) and FlowJo (FlowJo, LLC).

### *Viral fluorescence in situ hybridization (viralFISH) probe design, synthesis and application*

Probes were designed to target seven regions (300 bp each) of the OIV1 genome (GenBank accession HM004431.1) (Supporting Information Table S4). Probes were synthesized through PCR amplification via primer pairs and were incorporated with DIG‐dUTP using a PCR DIG Probe Synthesis Kit (Roche). Nonsense probe non‐Poly350Pr was used as a negative control gene probe (Moraru *et al.,* 2010; Dang *et al.,* 2015). The resulting PCR products (probes‐targets) were column‐purified using the Gene Clean Turbo Kit (Qbiogene, Inc.). Annealing/melting temperatures for probe‐target pairs were determined using real‐time PCR and SYTO 9 dye (Life Technologies).

ViralFISH was performed as previously described (Allers *et al.,* 2013), with some modifications. Briefly, samples from single biological replicates of OlV1‐infected cultures were spotted separately onto poly‐L‐lysin coated slides. Two slides were prepared for a non‐infected control culture from each SL and LL condition and prepared in the same manner as virus‐infected samples. After cell permeabilization and inactivation of endogenous peroxidases, eukaryotic 18S rRNA was detected with HRP‐conjugated oligonucleotide probes (Supporting Information Table S4, Integrated DNA Technologies), through a catalysed reporter deposition (CARD) reaction of Alexa488‐tyramides (Life Technologies). For detection of OIV1, samples were incubated with the Dig‐labelled probe mixtures, followed by binding of anti‐Dig HRP‐conjugated antibody (Fab fragments; Roche) and CARD of the Alexa594‐tyramides (Life Technologies). Slides were embedded in SlowFade antifade reagent (Invitrogen), containing DAPI. Microscopy was performed on Axioskop2 Mot Plus, equipped with Alexa488 (472/30 excitation, 520/35 emission, 495 Beam Splitter) and Alexa594 (562/40 excitation, 624/40 emission, 593 Beam Splitter) filter sets. A total of 200 infected cells were counted per sample and categorized into percentages of (i) virus‐attached cells, where viral signals are detected on the margin of host signals, (ii) infected cells, where virus and host signals overlap and (iii) virally lysed cells, where viral signals are concentrated around reduced or lost host signals.

### *DOM extraction, LC–MS/MS and spectral analysis*

DOM was extracted from <0.3 μm filtrates from samples collected at 24.5 h after addition of virus from each replicate of each treatment. DOM extraction procedures generally followed Dittmar *et al*. (2008). DOM samples were acidified to pH 2 before solid phase extraction using Agilent Bond Elut PPL cartridges (1 g, 6 ml). Cartridges were washed first with one column volume of methanol, equilibrated with one column volume of ultrapure water, followed by sample loading. After washing with two column volumes of 0.01 M HCl, DOM compounds sorbed to the column were eluted with 6 ml methanol. Eluate was then freeze‐dried (Centrivap, Labconco) and stored at −80°C.

Samples were re‐dissolved with 2% acetonitrile + 0.1% formic acid and 6 μl aliquots were injected onto a trapping column (OptiPak C18, Optimize Technologies) and separated on a capillary C18 column (Thermo Acclaim PepMap 100Å, 2 μm particles, 50 μm I.D. × 50 cm length) using a water‐acetonitrile + 0.1% formic acid gradient (2%–50% acetonitrile over 210 min) at 90 nl/min using a Dionex Ultimate 3000 LC system. Ionization was by nanoelectrospray (Proxeon Nanospray Flex) in positive mode. Mass spectra were collected on an Orbitrap Elite mass spectrometer (Thermo Fisher Scientific) operating in a data‐dependent acquisition (DDA) mode, with one high‐resolution (240,000 m/Δm at m/z 400) MS1 parent ion full scan triggering 15 MS2 Rapid mode CID fragment ion scans of intensity‐selected precursors.

Mass spectral data in Thermo RAW format were converted to mzXML format with ProteoWizard (Kessner *et al.,* 2008) and mzXML datafiles were uploaded to Global Natural Products Social Molecular Networking (GNPS) (Wang *et al.,* 2016). Spectral clustering in GNPS was based upon similarity cosine scoring of MS2 spectra. MS2 fragmentation patterns with sufficiently high cosine scores (≥0.7), and differences between parent masses' mass‐to‐charge ratio ≤100 Th, were grouped into the same spectral cluster. A Python data pipeline (available at https://github.com/WaldbauerLab/metabolomics; Ma *et al.,* 2018) was used to clean, transform and store spectral cluster data from GNPS, including merging spectral clusters which have parent masses within mass spectrometer analytical error (≤0.005 Da) and/or which appeared to be isotopologues. DOM mass spectral data are available via the MassIVE repository under dataset ID MSV000083434.

To test for differential abundance of spectral clusters between the three viral treatments (i.e., non‐infected control, OlV1‐infected, OlV7‐infected), we used DESeq2, an R statistical package (Love *et al.,* 2014) that enables quantification and statistical inference of systematic changes between experimental conditions with a wide variety of discrete data types. Here, we applied it for differential analysis of GNPS‐clustered mass spectrometric data, including three pairwise comparisons: OlV1‐infected vs. non‐infected control, OlV7‐infected vs. non‐infected control and OlV1‐infected vs. OlV7‐infected; data from SL and LL conditions of each infection treatment were combined to improve the sample size (from *n* = 3 to *n* = 6) and statistical rigour for these analyses. Spectral clusters with *P* values ≤0.01 against the null hypothesis of equal abundance in each pairwise condition comparison were considered significantly differentially abundant. We further assessed the differential abundance of this subset of spectral clusters between SL and LL conditions within each infection treatment (i.e., OlV1 SL vs. LL, OlV7 SL vs. LL, non‐infected control SL vs. LL).

We attempted to identify putative compounds represented by the differentially abundant spectral clusters using the approaches described previously (Ma *et al.,* 2018). Briefly, ChemCalc (Krompiec and Patiny, 2001) and MetFrag (Ruttkies *et al.,* 2016) were used to predict compound stoichiometry and compare spectral cluster fragmentation patterns to known compounds from molecular structure databases. Mass spectral data were also searched against a protein database generated from the *O. lucimarinus* CCMP2972 and OlV genomes to attempt to identify spectral clusters with peptide‐like features using SEQUEST HT implemented in Proteome Discoverer (Thermo Scientific), with unspecific protein cleavage and controlling peptide‐ and protein‐level FDRs to 0.01 using Percolator.

### *Calculations and statistical analysis*

The temporal dynamics of virus and host populations were assessed by the log2 fold change in abundance at each time point, which is equivalent to the number of generations (*n*) during exponential growth. Log2 fold change was calculated as: *n*_*t*_ = (ln(*N*_*t*_) − ln(*N*_*i*_))/*ln*(2), where *N*_*t*_ is the number of virions or host cells at time *t*, and *N*_*i*_ is the number of virions or host cells at the initial time point [*i* = −3.5 h (i.e., dawn) for host cells and 0.5 h for virions]. Specific host growth rate (*μ*) was calculated as: *μ* = ln(*N*_*t*_/*N*_*i*_)/(Δ*t*).

Viral latent period was calculated as the lapse‐time between addition of viruses to the host culture and the time point at which the log2 fold change in virus abundance was significantly greater than zero. Due to the difference in starting inoculum of OlV1 (8.19 ± 0.82 × 10^7^ ml^−1^) and OlV7 (1.75 ± 0.18 × 10^7^ ml^−1^), viral production (VP) was estimated as the proportional (fold) change in virion abundance at each time point: VP = *N*_*t*_/*N*_*i*_. This metric was used to evaluate the response of viral production to irradiance condition. An approximation of viral burst size was calculated from the increase in virions and the loss of host cells between 18.5 and 24.5 h after addition of virus.

Intracellular dynamics during the infection cycle were estimated as follows: *F*_I_ = *F* ^>G1^ − *F* ^D^, *F* ^D^ = *F* ^D^_C_ × *s*, *s* = *n*_I_/*n*_C_, where *F*_I_ represents the frequency of infected host cells, *F* ^>G1^ represents the frequency of host cells with a SYBR fluorescence (i.e., DNA) signal great than G1‐phase cells, *F* ^D^ represents the frequency of dividing host cells (i.e., cells in S, G2 or M phases), *F* ^D^_C_ represents the mean frequency of dividing cells in the corresponding non‐infected control cultures, and *s* represents a scaling factor based on the relative number of generations (*n*) in corresponding infected and non‐infected control cultures. Instances where *s* < 0 or *s* > 1 were treated as *s* = 0 or *s* = 1 respectively. Note that *F* ^>G1^ = *F* ^D^ for non‐infected control cultures.

Significant effects of viral treatment (levels = no virus, +OlV1, or +OlV7) were tested with one‐way analysis of variance (ANOVA) at each time point using Type II Sum of Squares, followed by Tukey‐adjusted pairwise comparisons. ANOVA and adjusted post hoc comparisons were also used to assess the variance across time points for each virus X irradiance condition combination. Comparisons between irradiance conditions (LL vs. SL) and viruses (OlV1 vs. OlV7) were conducted with Welch's two‐sample *t*‐tests assuming unequal variances. Significance was evaluated as *P* < 0.05, unless otherwise noted. All tests were implemented within R (R Core Team, 2017), using the default ‘stats’ package, ‘car’ package (Fox and Weisberg, 2011) or ‘lsmeans’ package (Lenth, 2016).

## Supporting information

As per Mahmoudabadi *et al*. (2017), the direct energetic cost of genome replication for a virus with a dsDNA genome was approximated as: *E*_REP(dsDNA)/v_ ≈ 2*L*_g_(*e*_d_ + *e*_p_), where *L*_g_ represents to the genome length and is multiplied by two to account for a double‐stranded genome. The cost of each DNA nucleotide in the genome is calculated as the sum of *e*_d_, which represents the average direct cost of DNA synthesis from precursor metabolites (estimated as 11 ATP‐equivalent hydrolysis events; see Dataset S1 in Mahmoudabadi *et al.,* 2017 for a detailed derivation of this cost), and *e*_p_, which denotes the cost of chain elongation per base (estimated as two ATP‐equivalent hydrolysis events during viral synthesis; Lynch and Marinov, 2015).

## Supporting information


**Appendix S1.** Supporting information

**Table S1.** Genome characteristics of *Ostreococcus lucimarinus* viruses 1 and 7.
**Table S2.** Orthologous protein‐coding genes in OlV1 and OlV7.
**Table S3.** Protein‐coding genes specific to OlV1 or OlV7 and orthologues found in other prasinoviruses.
**Table S4.** Primer and probe oligonucleotide sequences used for viralFISH
**Table S5.** Summary of the numbers of MS2 spectra from DOM analysis.

**Figure S1.** Cellular characteristics of *O. lucimarinus* over the infection cycle for cultures acclimated to 105‐115 μmol photons m^−2^ s^−2^ irradiance (SL) (A), or 15 μmol photons m^−2^ s^−2^ irradiance (LL) (B). Mean forward angle light scatter (FALS) in bead relative units (i.e., normalized to beads) approximates cell size. Non‐infected control (open circles), OlV1‐ (black circles), and OlV7‐ (grey circles) infected treatments are shown. Points show mean ± standard deviation of biological replicates (n = 3). Shaded areas indicate dark period in 14:10 hour diel cycle.

**Figure S2.** Growth of host cultures shifted to 15 μmol photons m^−2^ s^−2^ irradiance (0.091 ± 0.082 d^−1^ growth rate at time of infection) and viral life cycle of OlV1 and OlV7 resolved by analytical flow cytometry. (A) Growth curves of algal hosts without viruses (open circles) and with addition of OlV1 (black circles) or OlV7 (grey circles), shown as the log2 fold change in abundance (equivalent to number of generations during exponential growth) since dawn (T = ‐4 h). Statistical tests showed that the reduction in light from SL to LL conditions reduced the growth rate significantly (Welch's two‐sample t‐test, *P* < 0.01) within 2 days. (B) OlV1 (black triangles) and OlV7 (grey triangles) abundance over the infection cycle shown as the log2 fold change relative to the time viruses were added to cultures (T = 0 h). (C) Percentages of algal cells that were actively dividing (sum of cells in S, G2, or M phases) as inferred from cell cycle analysis of SYBR‐stained samples. The growth of OlV1‐ and OlV7‐infected cultures relative to non‐infected cultures in panel A were used to calculate the percentages of dividing cells in infected cultures at each time point from non‐infected culture values (see methods for more details). (D) The percentages of infected host cells were inferred from SYBR‐stained samples, after accounting for cells in S, G2, and M phases of the cell cycle. Points show mean ± standard deviation of biological replicates (n = 3). Shaded areas indicate dark period in 14:10 hour diel cycle.

**Figure S3.** Progression of OlV1 viral infection over time resolved by viralFISH. A total of 200 infected cells were counted per sample and categorized into percentages of (i) virus‐attached cells (light grey), where viral signals are detected on the margin of host signals, (ii) infected cells (dark grey), where virus and host signals overlap, (iii) virally‐lysed cells (black), where viral signals are concentrated around reduced or lost host signals, or (iv) non‐infected cells (white), where no viral signals were detected with host signals. Progression of infection was evaluated in (A) SL (105‐115 μmol photons m^−2^ s^−2^, 0.76 ± 0.06 d^−1^ growth rate at time of infection) and (B) LL irradiance (15 μmol photons m^−2^ s^−2^, 0.091 ± 0.082 d^−1^ growth rate at time of infection). Values determined from single biological replicates (n = 1). Shaded areas indicate dark period in 14:10 hour diel cycle.

**Figure S4.** Growth rates of non‐infected *O. lucimarinus* in experimental flasks at two irradiance levels: (A) 105‐115 μmol photons m^−2^ s^−2^ (SL), or (B) 15 μmol photons m^−2^ s^−2^ irradiance (LL). Growth rates were calculated for each 24‐hour interval. Shading was added to reduce irradiance at T = ‐48 h. OlV1 and OlV7 were added to infected treatments (not shown) at T = 0 h (dashed line). Points show mean ± standard deviation of biological replicates (n = 3). Shaded areas indicate dark period in 14:10 hour diel cycle.

**Figure S5.** Representative flow cytometry histograms of SYBR green fluorescence (i.e., relative DNA content) over the infection cycle for non‐infected control, OlV1‐infected, and OlV7‐infected cultures acclimated to 105‐115 μmol photons m^−2^ s^−2^ irradiance (SL). The gate of the G1‐phase *O. lucimarinus* host population is shown (blue bar). Both x‐ and y‐axes are plotted on a linear scale. The range of the x‐axis is consistent across all panels. The maximum value of the y‐axis differs across treatments, such that y_max_ of non‐infected controls is 770 (top row), y_max_ of OlV1‐infected cultures is 30 (middle row), and y_max_ of OlV7‐infected cultures is 40 (bottom row).

**Figure S6.** Viral infection of *O. lucimarinus* by OlV1 or OlV7 grown under 105‐115 μE m^−2^ s^−2^ irradiance (SL, A and C) or 15 μE m^−2^ s^−2^ irradiance (LL, B and D). Dynamics of host abundance are shown in the upper panels (A and B) for non‐infected control (open circles), OlV1‐ (black circles), and OlV7‐infected (grey circles) treatments. Dynamics of virus abundance are shown in the bottom panels (C and D) for OlV1 (black triangles) and OlV7 (grey triangles). Note that a greater abundance of OlV1 virions was added at T = 0 h to account for lower infectivity as compared to OlV7 (Table 1). Points show mean ± standard deviation of biological replicates (n = 3). Shaded areas indicate dark period in 14:10 hour diel cycle.
