# Supplementary material for: Closely related viruses of the marine picoeukaryotic alga Ostreococcus lucimarinus exhibit different ecological strategies
Source: Environ Microbiol. 2019 May 13;21(6):2148–70. doi: 10.1111/1462-2920.14608 (PMC6851583; doi:10.1111/1462-2920.14608)
Supplement: Supplementary file 1 — Appendix S1. Supporting information [file EMI-21-2148-s001.docx]

For re-submission to *Environmental Microbiology*

*Special Issue on Viruses*

Supporting Information for

**Closely-related viruses of the marine picoeukaryotic alga *Ostreococcus lucimarinus* exhibit different ecological strategies**

Amy E. Zimmerman^1,a^, Charles Bachy^1,b^, Xiufeng Ma^2,c^, Simon Roux^3.d^, Ho Bin Jang^3,4^, Matthew B. Sullivan^3,4^, Jacob R. Waldbauer^2^, Alexandra Z. Worden*^1,5^

^1^Monterey Bay Aquarium Research Institute, Moss Landing, CA, USA

^2^Department of the Geophysical Sciences, University of Chicago, Chicago, IL, USA

^3^Department of Microbiology, ^4^Department of Civil, Environmental and Geodetic Engineering, The Ohio State University, Columbus, OH, USA

^5^Ocean EcoSystems Biology Unit, Marine Ecology Division, GEOMAR Helmholtz Centre for Ocean Research Kiel, Kiel, DE

**SUPPORTING INFORMATION**

As per Mahmoudabadi et al*.* (2017), the direct energetic cost of genome replication for a virus with a dsDNA genome was approximated as: *E_REP_*_(_*_dsDNA_*_)/_*_v_* ≈ 2*L_g_*(*e_d_* + *e_p_*) where *L_g_* represents to the genome length and is multiplied by 2 to account for a double-stranded genome. The cost of each DNA nucleotide in the genome is calculated as the sum of *e_d_*, which represents the average direct cost of DNA synthesis from precursor metabolites (estimated as 11 ATP-equivalent hydrolysis events; see Dataset S1 in Mahmoudabadi et al., 2017 for a detailed derivation of this cost), and *e_p_*, which denotes the cost of chain elongation per base (estimated as 2 ATP-equivalent hydrolysis events during viral synthesis; Lynch and Marinov, 2015).

**REFERENCES**

Lynch, M. and Marinov, G.K. (2015) The bioenergetic costs of a gene. *Proc. Natl. Acad. Sci.* **112**: 15690–15695.

Mahmoudabadi, G., Milo, R., and Phillips, R. (2017) The Energetic Cost of Building a Virus. *Proc. Natl. Acad. Sci.* **114**: E4324–E4333.

**SUPPLEMENTARY TABLES**

Please see corresponding spreadsheets.

Table S1: Genome characteristics of *Ostreococcus lucimarinus* viruses 1 and 7.

Table S2: Orthologous protein-coding genes in OlV1 and OlV7.

Table S3: Protein-coding genes specific to OlV1 or OlV7 and orthologues found in other prasinoviruses.

Table S4: Primer and probe oligonucleotide sequences used for viralFISH

Table S5: Summary of the numbers of MS2 spectra from DOM analysis.

**SUPPLEMENTARY FIGURE LEGENDS**

**Figure S1.** Cellular characteristics of *O. lucimarinus* over the infection cycle for cultures acclimated to 105-115 µmol photons m^-2^ s^-2^ irradiance (A), or 15 µmol photons m^-2^ s^-2^ irradiance (B). Mean forward angle light scatter (FALS) in bead relative units (i.e., normalized to beads) approximates cell size. Non-infected control (open circles), OlV1- (black circles), and OlV7-infected treatments are shown. Points show mean ± standard deviation of biological replicates (n=3). Shaded areas indicate dark period in 14:10 hour diel cycle.

**Figure S2.** Growth of host cultures shifted to 15 µmol photons m^-2^ s^-2^ irradiance (0.091±0.082 d^-1^ growth rate at time of infection) and viral life cycle of OlV1 and OlV7 resolved by analytical flow cytometry. (A) Growth curves of algal hosts without viruses (open circles) and with addition of OlV1 (black circles) or OlV7 (gray circles), shown as the log2 fold change in abundance (equivalent to number of generations during exponential growth) since dawn (T-4 hrs). Statistical tests showed that the reduction in light from SL to LL conditions reduced the growth rate significantly (Welch’s two-sample t-test, *P*<0.01) within 2 days. (B) OlV1 (black triangles) and OlV7 (gray triangles) abundance over the infection cycle shown as the log2 fold change relative to the time viruses were added to cultures (T=0 hrs). (C) Percentages of algal cells that were actively dividing (sum of cells in S, G2, or M phases) as inferred from cell cycle analysis of SYBR-stained samples. The growth of OlV1- and OlV7-infected cultures relative to non-infected cultures in panel A were used to calculate the percentages of dividing cells in infected cultures at each time point from non-infected culture values (see methods for more details). (D) The percentages of infected host cells were inferred from SYBR-stained samples, after accounting for cells in S, G2, and M phases of the cell cycle. Points show mean ± standard deviation of biological replicates (n=3). Shaded areas indicate dark period in 14:10 hour diel cycle.

**Figure S3.** Progression of OlV1 viral infection over time resolved by viralFISH. A total of 200 infected cells were counted per sample and categorized into percentages of (i) virus-attached cells (light gray), where viral signals are detected on the margin of host signals, (ii) infected cells (dark gray), where virus and host signals overlap, (iii) virally-lysed cells (black), where viral signals are concentrated around reduced or lost host signals, or (iv) non-infected cells (white), where no viral signals were detected with host signals. Progression of infection was evaluated in (A) SL (105-115 µmol photons m^-2^ s^-2^, 0.76±0.06 d^-1^ growth rate at time of infection) and (B) LL irradiance (15 µmol photons m^-2^ s^-2^, 0.091±0.082 d^-1^ growth rate at time of infection). Values determined from single biological replicates (n=1). Shaded areas indicate dark period in 14:10 hour diel cycle.

**Figure S4.** Growth rates of non-infected *O. lucimarinus* in experimental flasks at two irradiance levels: (A) 105-115 µmol photons m^-2^ s^-2^, or (B) 15 µmol photons m^-2^ s^-2^ irradiance (LL). Growth rates were calculated for each 24-hour interval. Shading was added to reduce irradiance at T-48 hrs. OlV1 and OlV7 were added to infected treatments (not shown) at T0 hrs (dashed line). Points show mean ± standard deviation of biological replicates (n=3). Shaded areas indicate dark period in 14:10 hour diel cycle.

**Figure S5.** Representative flow cytometry histograms of SYBR green fluorescence (i.e., relative DNA content) over the infection cycle for non-infected control, OlV1-infected, and OlV7-infected cultures acclimated to 105-115 µmol photons m^-2^ s^-2^ irradiance (SL). The gate of the G1-phase *O. lucimarinus* host population is shown (blue bar). Both x- and y-axes are plotted on a linear scale. The range of the x-axis is consistent across all panels. The maximum value of the y-axis differs across treatments, such that y_max_ of non-infected controls is 770 (top row), y_max_ of OlV1-infected cultures is 30 (middle row), and y_max_ of OlV7-infected cultures is 40 (bottom row).

**Figure S6.** Viral infection of *O. lucimarinus* by OlV1 or OlV7 grown under 105-115 µE m^-2^ s^-2^ irradiance (SL, A and C) or 15 µE m^-2^ s^-2^ irradiance (LL, B and D). Dynamics of host abundance are shown in the upper panels (A and B) for non-infected control (open circles), OlV1- (black circles), and OlV7-infected (gray circles) treatments. Dynamics of virus abundance are shown in the bottom panels (C and D) for OlV1 (black triangles) and OlV7 (gray triangles). Note that a greater abundance of OlV1 virions was added at T0 hrs to account for lower infectivity as compared to OlV7 (Table 1). Points show mean ± standard deviation of biological replicates (n=3). Shaded areas indicate dark period in 14:10 hour diel cycle.
