# Supplementary figures and images for: Closely related viruses of the marine picoeukaryotic alga Ostreococcus lucimarinus exhibit different ecological strategies
Source: Environ Microbiol. 2019 May 13;21(6):2148–70. doi: 10.1111/1462-2920.14608 (PMC6851583; doi:10.1111/1462-2920.14608)

**A**

Normalized FALS

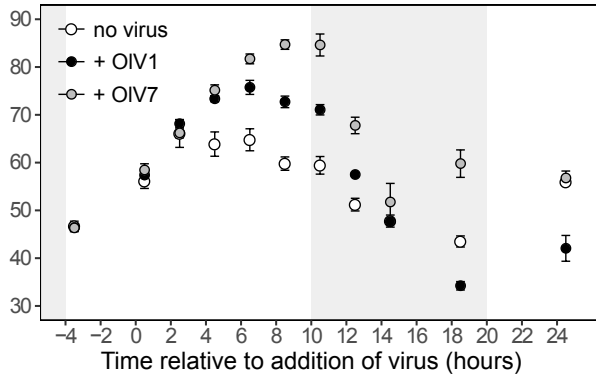**B**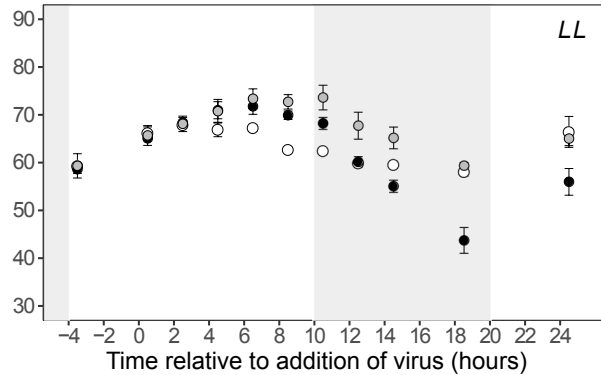

Supplement: Supplementary file 3 — Figure S1. Cellular characteristics of O. lucimarinus over the infection cycle for cultures acclimated to 105‐115 μmol photons m−2 s−2 irradiance (SL) (A), or 15 μmol photons m−2 s−2 irradiance (LL) (B). Mean forward angle light scatter (FALS) in bead relative units (i.e., normalized to beads) approximates cell size. Non‐infected control (open circles), OlV1‐ (black circles), and OlV7‐ (grey circles) infected treatments are shown. Points show mean ± standard deviation of biological replicates (n = 3). Shaded areas indicate dark period in 14:10 hour diel cycle. [file EMI-21-2148-s003.pdf]

**A**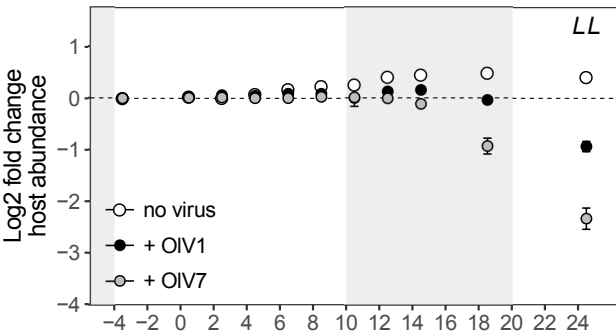**B**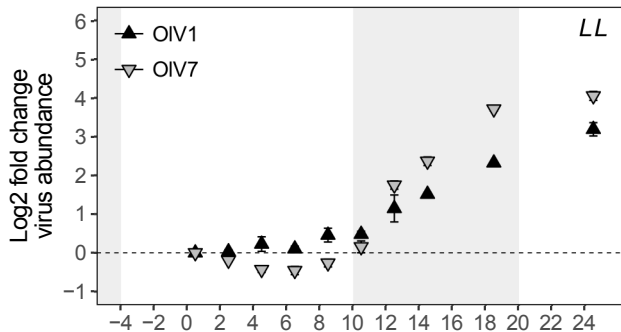**C**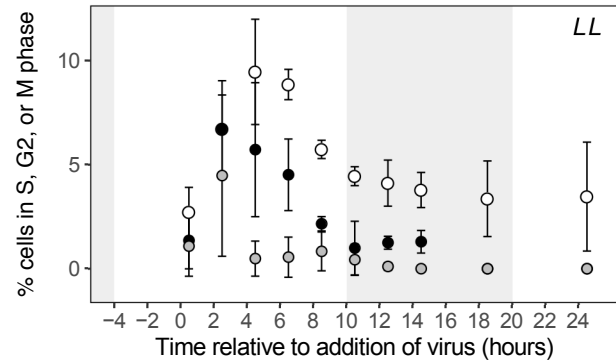**D**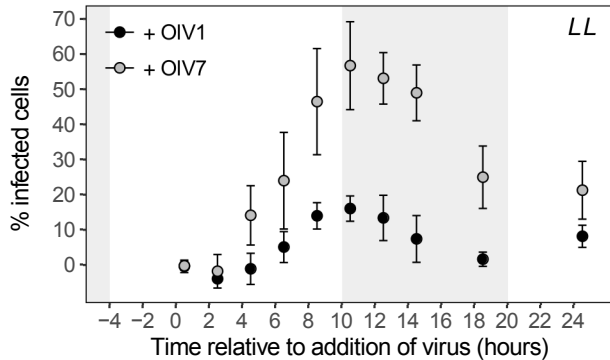

Supplement: Supplementary file 4 — Figure S2. Growth of host cultures shifted to 15 μmol photons m−2 s−2 irradiance (0.091 ± 0.082 d−1 growth rate at time of infection) and viral life cycle of OlV1 and OlV7 resolved by analytical flow cytometry. (A) Growth curves of algal hosts without viruses (open circles) and with addition of OlV1 (black circles) or OlV7 (grey circles), shown as the log2 fold change in abundance (equivalent to number of generations during exponential growth) since dawn (T = ‐4 h). Statistical tests showed that the reduction in light from SL to LL conditions reduced the growth rate significantly (Welch's two‐sample t‐test, P < 0.01) within 2 days. (B) OlV1 (black triangles) and OlV7 (grey triangles) abundance over the infection cycle shown as the log2 fold change relative to the time viruses were added to cultures (T = 0 h). (C) Percentages of algal cells that were actively dividing (sum of cells in S, G2, or M phases) as inferred from cell cycle analysis of SYBR‐stained samples. The growth of OlV1‐ and OlV7‐infected cultures relative to non‐infected cultures in panel A were used to calculate the percentages of dividing cells in infected cultures at each time point from non‐infected culture values (see methods for more details). (D) The percentages of infected host cells were inferred from SYBR‐stained samples, after accounting for cells in S, G2, and M phases of the cell cycle. Points show mean ± standard deviation of biological replicates (n = 3). Shaded areas indicate dark period in 14:10 hour diel cycle. [file EMI-21-2148-s004.pdf]

**A**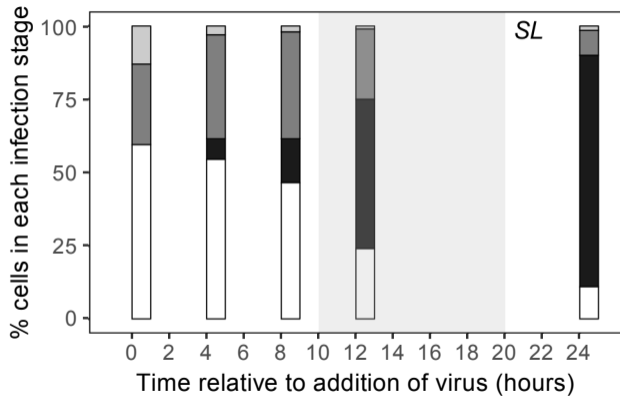**B**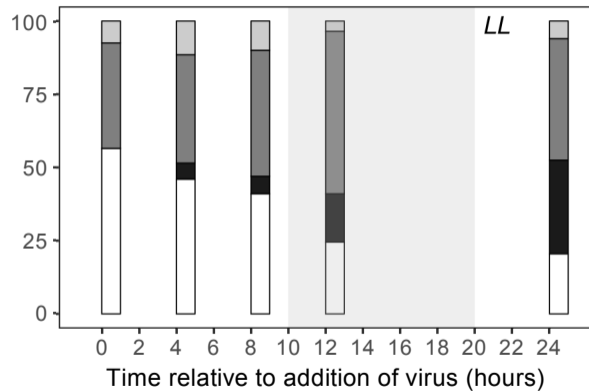

Attached    Infected    Lysed    Noninfected

Supplement: Supplementary file 5 — Figure S3. Progression of OlV1 viral infection over time resolved by viralFISH. A total of 200 infected cells were counted per sample and categorized into percentages of (i) virus‐attached cells (light grey), where viral signals are detected on the margin of host signals, (ii) infected cells (dark grey), where virus and host signals overlap, (iii) virally‐lysed cells (black), where viral signals are concentrated around reduced or lost host signals, or (iv) non‐infected cells (white), where no viral signals were detected with host signals. Progression of infection was evaluated in (A) SL (105‐115 μmol photons m−2 s−2, 0.76 ± 0.06 d−1 growth rate at time of infection) and (B) LL irradiance (15 μmol photons m−2 s−2, 0.091 ± 0.082 d−1 growth rate at time of infection). Values determined from single biological replicates (n = 1). Shaded areas indicate dark period in 14:10 hour diel cycle. [file EMI-21-2148-s005.pdf]

**A**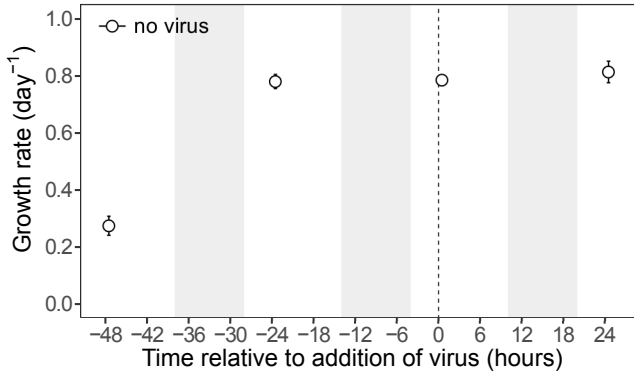**B**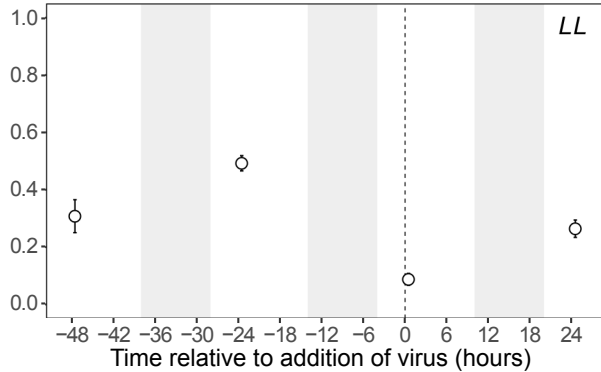

Supplement: Supplementary file 6 — Figure S4. Growth rates of non‐infected O. lucimarinus in experimental flasks at two irradiance levels: (A) 105‐115 μmol photons m−2 s−2 (SL), or (B) 15 μmol photons m−2 s−2 irradiance (LL). Growth rates were calculated for each 24‐hour interval. Shading was added to reduce irradiance at T = ‐48 h. OlV1 and OlV7 were added to infected treatments (not shown) at T = 0 h (dashed line). Points show mean ± standard deviation of biological replicates (n = 3). Shaded areas indicate dark period in 14:10 hour diel cycle. [file EMI-21-2148-s006.pdf]

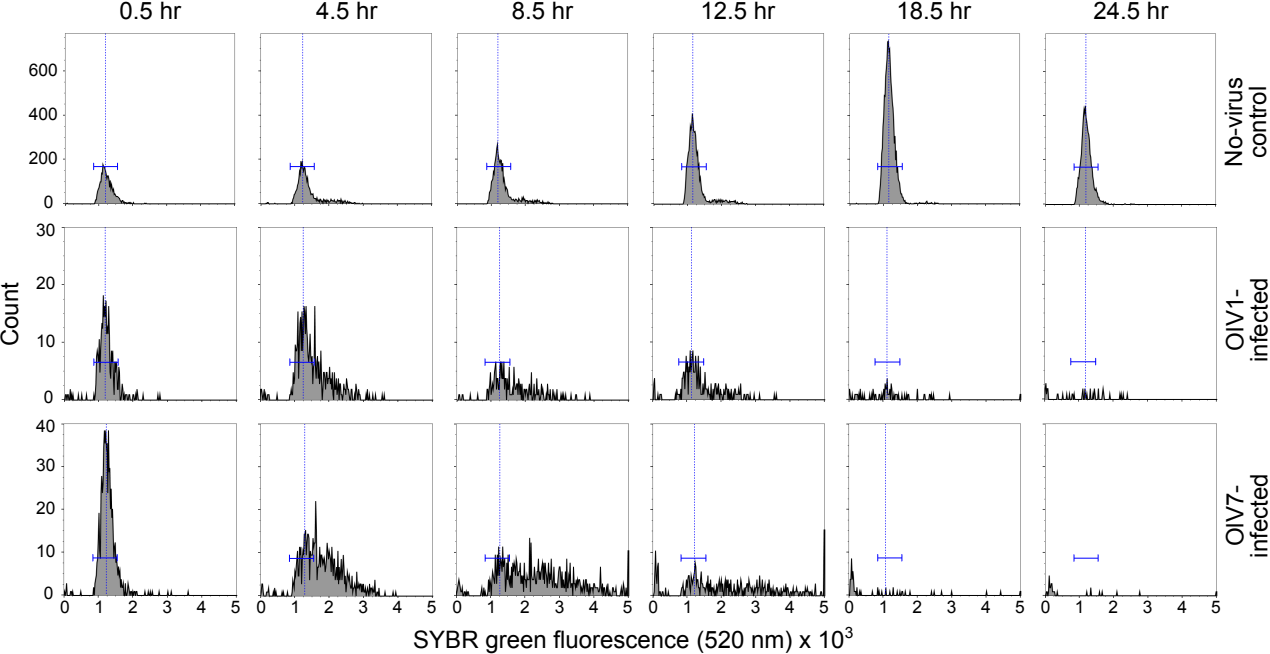

Supplement: Supplementary file 7 — Figure S5. Representative flow cytometry histograms of SYBR green fluorescence (i.e., relative DNA content) over the infection cycle for non‐infected control, OlV1‐infected, and OlV7‐infected cultures acclimated to 105‐115 μmol photons m−2 s−2 irradiance (SL). The gate of the G1‐phase O. lucimarinus host population is shown (blue bar). Both x‐ and y‐axes are plotted on a linear scale. The range of the x‐axis is consistent across all panels. The maximum value of the y‐axis differs across treatments, such that ymax of non‐infected controls is 770 (top row), ymax of OlV1‐infected cultures is 30 (middle row), and ymax of OlV7‐infected cultures is 40 (bottom row). [file EMI-21-2148-s007.pdf]

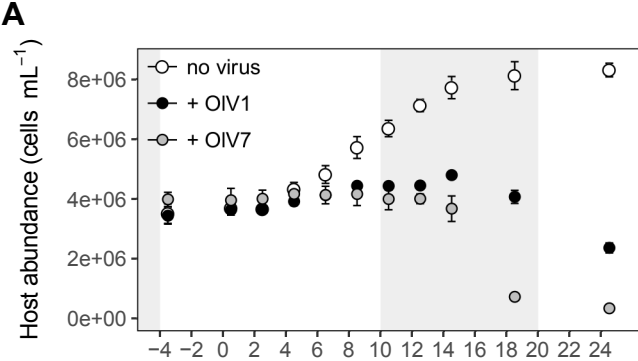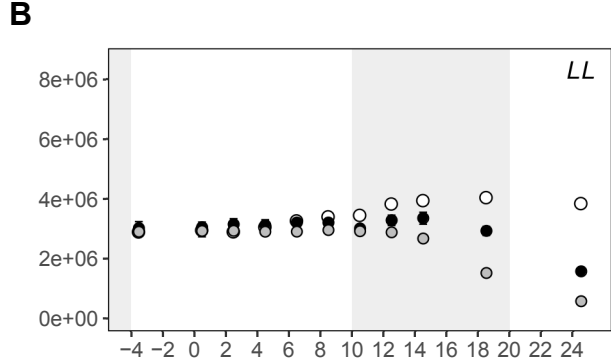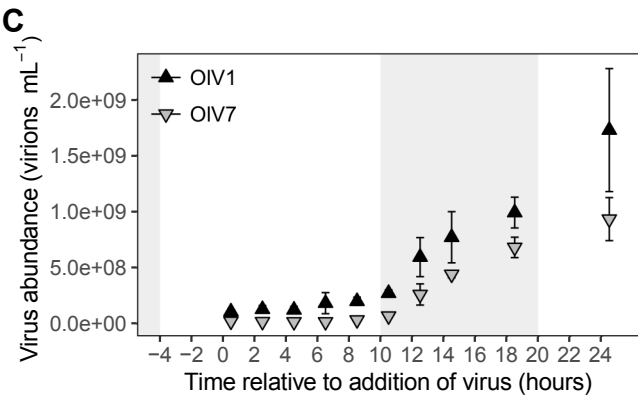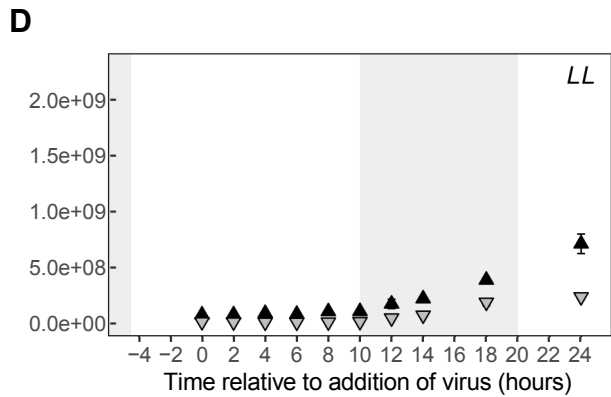

Supplement: Supplementary file 8 — Figure S6. Viral infection of O. lucimarinus by OlV1 or OlV7 grown under 105‐115 μE m−2 s−2 irradiance (SL, A and C) or 15 μE m−2 s−2 irradiance (LL, B and D). Dynamics of host abundance are shown in the upper panels (A and B) for non‐infected control (open circles), OlV1‐ (black circles), and OlV7‐infected (grey circles) treatments. Dynamics of virus abundance are shown in the bottom panels (C and D) for OlV1 (black triangles) and OlV7 (grey triangles). Note that a greater abundance of OlV1 virions was added at T = 0 h to account for lower infectivity as compared to OlV7 (Table 1). Points show mean ± standard deviation of biological replicates (n = 3). Shaded areas indicate dark period in 14:10 hour diel cycle. [file EMI-21-2148-s008.pdf]
